# Large-scale outdoor 3D trajectory measurements enabled by drone-assisted multicamera tracking

**DOI:** 10.1126/sciadv.aeh8352

**Published:** 2026-08-19

**Authors:** Jungyeon Kim, Sangmin Lim, Pius Soh, Jin-Tae Kim

**Affiliations:** Department of Mechanical Engineering, Pohang University of Science and Technology, Pohang 37673, Republic of Korea.

## Abstract

Accurate measurement of three-dimensional (3D) trajectories in outdoor environments is essential for studying complex dynamic processes such as atmospheric transport, aerial vehicle motion, and collective behavior. However, obtaining quantitative trajectory measurements over large outdoor volumes remains challenging because multicamera systems require reliable calibration and synchronization under field conditions. Here, we introduce a portable and automated drone-assisted multicamera 3D tracking (DAM3T) framework for georeferenced trajectory reconstruction. The system estimates multicamera extrinsic parameters from an autonomous drone flight without dedicated calibration markers and aligns the reconstructed coordinate system with geographic coordinates (WGS84) using real-time kinematic Global Positioning System (RTK-GPS) measurements. Validation under indoor motion-capture and outdoor RTK-GPS conditions demonstrates trajectory errors below 2%. We further demonstrate the framework through Lagrangian analysis using passive tracers, georeferenced tracking of multiple unmanned aerial vehicles, and 3D motion analysis in sports scenes. This establishes DAM3T as a scalable measurement platform that extends outdoor tracking from local-coordinate reconstruction to georeferenced measurement of complex dynamic systems.

## INTRODUCTION

Accurate measurement of three-dimensional (3D) trajectories of moving targets in outdoor environments is essential for understanding complex dynamic processes in natural and engineering systems. Examples include atmospheric transport of particles and pollutants (*1*–*3*), turbulence-induced hazards near airports (*4*, *5*), dispersion of smoke and debris during wildfires (*6*, *7*), and collective search behavior in bats (*8*). In these scenarios, the ability to reconstruct trajectories over large spatial scales is critical for linking observed motion to the underlying flow physics and environmental conditions. Achieving such measurements in field environments remains challenging, however, because existing sensing technologies struggle to provide accurate, scalable, and rapidly deployable 3D tracking over large outdoor volumes. Recent studies have explored several approaches for outdoor 3D measurements, including multicamera tracking systems, RGB-D (red, green, blue, and depth) sensing that combines RGB cameras with depth sensors, and Doppler light detection and ranging (LiDAR). Representative examples include flow analysis through tracking of natural particles using multicamera observations (*1*, *2*), 3D reconstruction of smoke using coordinated drone camera swarms (*6*), measurement of drone wakes under outdoor conditions using Doppler LiDAR (*9*), and LiDAR-based tracking of unmanned aerial vehicles (UAVs) (*10*). Although these methods demonstrate the potential of outdoor sensing technologies, practical field deployment remains challenging.

A major difficulty arises from the calibration and synchronization requirements of large-scale sensing systems. Outdoor scenes often exhibit strong variability in illumination and background appearance across large measurement volumes, which complicates the establishment of reliable calibration pipelines for multicamera or RGB-D systems. To address this challenge, a variety of calibration strategies has been proposed, including wand-based procedures that exploit known length and gravity-direction constraints (*11*), drone-mounted dual light-emitting diodes (LEDs) that emulate a calibration wand (*1*, *2*), drone trajectory–based approaches that jointly estimate time synchronization and camera extrinsic parameters (*12*–*15*), calibration using an AprilTag-equipped drone and Global Positioning System (GPS) waypoint coordinates (*16*), camera systems equipped with inertial measurement units to estimate camera poses (*17*, *18*), vehicle-based calibration methods for urban CCTV (closed-circuit television) networks using GPS and 3D markers (*19*, *20*), targetless approaches based on background features with known coordinates (*21*, *22*), and parabolic trajectory–based methods that jointly estimate time synchronization and camera calibration using a parametric curve model (*23*, *24*). Despite these advances, existing approaches often face practical limitations for field deployment, including synchronization errors, limited aerial coverage, manual marker placement, or incomplete integration of georeferenced alignment within the calibration workflow. As a result, establishing a portable and automated calibration pipeline remains a key challenge for large-scale outdoor measurements.

Here, a drone-assisted multicamera 3D tracking (DAM3T) framework is introduced as a field-deployable system for outdoor 3D measurement systems. The approach uses an unmodified drone as a dynamic calibration target, enabling shared geometric constraints to be established across large measurement volumes through the observed drone trajectory. The trajectory is automatically detected and tracked in each camera view, allowing reconstruction of a consistent multicamera coordinate system. This reconstructed coordinate system is subsequently aligned with a georeferenced frame by registering the estimated trajectory to real-time kinematic GPS (RTK-GPS) measurements used as ground truth (GT). Unlike conventional visual tracking systems that reconstruct motion in an arbitrary local coordinate system, DAM3T uses drone calibration with RTK-GPS measurements to produce georeferenced multicamera visual measurements. This allows reconstructed trajectories to be interpreted in real-world coordinates. By integrating large-scale multicamera calibration with georeferenced alignment into a single automated workflow, DAM3T provides a portable measurement platform for outdoor trajectory reconstruction and enables quantitative studies of aerial vehicle dynamics, atmospheric flow phenomena, and multiagent motion in field environments. The performance of the system is validated using indoor motion-capture and outdoor RTK-GPS measurements, followed by demonstrations in multi-UAV tracking, passive tracer–based Lagrangian flow analysis, and multiagent motion tracking in sports scenes.

## RESULTS

### System overview

**Fig. 1.**
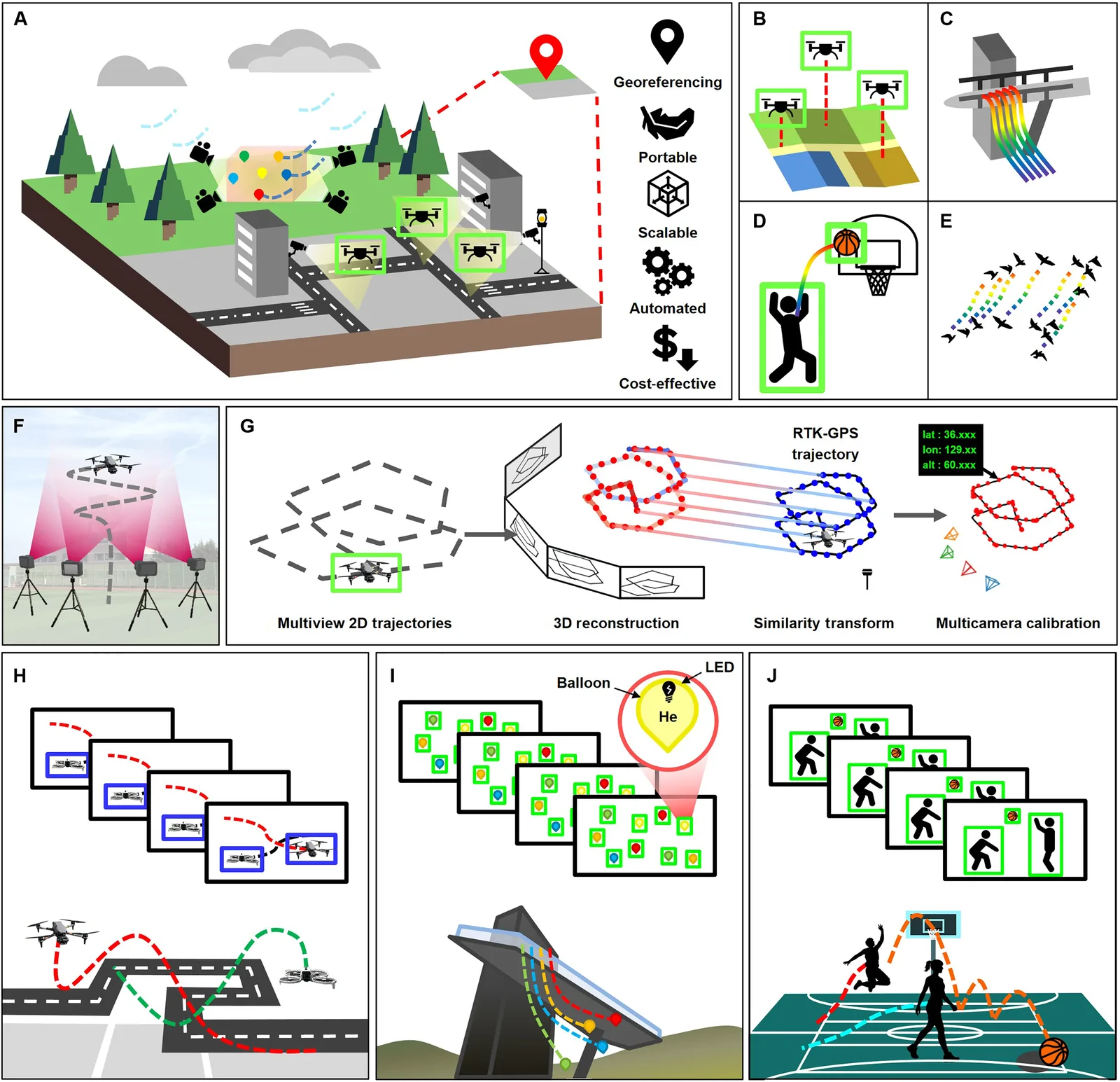
Overview of the DAM3T framework for georeferenced outdoor 3D tracking. (**A**) Conceptual illustration of the DAM3T system for outdoor 3D flow measurement and 3D UAV tracking in field environments. (**B** to **E**) Representative tracking scenarios enabled by DAM3T, including (B) multiple UAV trajectory tracking, (C) Lagrangian tracer measurements for flow visualization, (D) tracking of basketballs and players in sports scenes, and (E) collective motion tracking of bird swarms. (**F**) Example field setup. (**G**) Multicamera calibration pipeline. (**H** to **J**) Applications: (H) active object identification and trajectory reconstruction, (I) passive tracer tracking using LED-illuminated helium balloons for flow measurements, and (J) multiobject tracking in sports scenarios.

DAM3T is a portable, scalable, automated, and cost-effective field measurement system designed for georeferenced 3D tracking in outdoor environments (Fig. 1A). The framework enables trajectory reconstruction for diverse moving targets, including multiple UAVs (Fig. 1B) (*25*), Lagrangian tracers for flow analysis (Fig. 1C) (*1*, *2*), ball-sport scenarios involving players and a ball (Fig. 1D) (*26*), and collective motion such as bird swarms (Fig. 1E) (*11*). The system combines a calibration drone and a synchronized multicamera array (Fig. 1F). During calibration, the drone follows a predefined autonomous flight path, generating a dynamic trajectory that is simultaneously observed by all cameras. This shared trajectory serves as a calibration reference for estimating camera extrinsic parameters (Fig. 1G). This strategy is particularly advantageous for outdoor environments where reliable background features are often absent and deploying large calibration targets across the measurement volume is impractical. By aligning the reconstructed drone trajectory with RTK-GPS measurements, the system enables georeferenced multicamera calibration in large outdoor scenes. This allows reconstructed 3D points to be mapped to latitude, longitude, and altitude coordinates. Once calibrated, DAM3T supports multiple tracking modes including active object identification (Fig. 1H), passive tracer tracking (Fig. 1I), and multiobject tracking in sports scenes (Fig. 1J). In each case, synchronized two-dimensional (2D) observations from multiple cameras are triangulated to reconstruct 3D trajectories.

### System validation

**Fig. 2.**
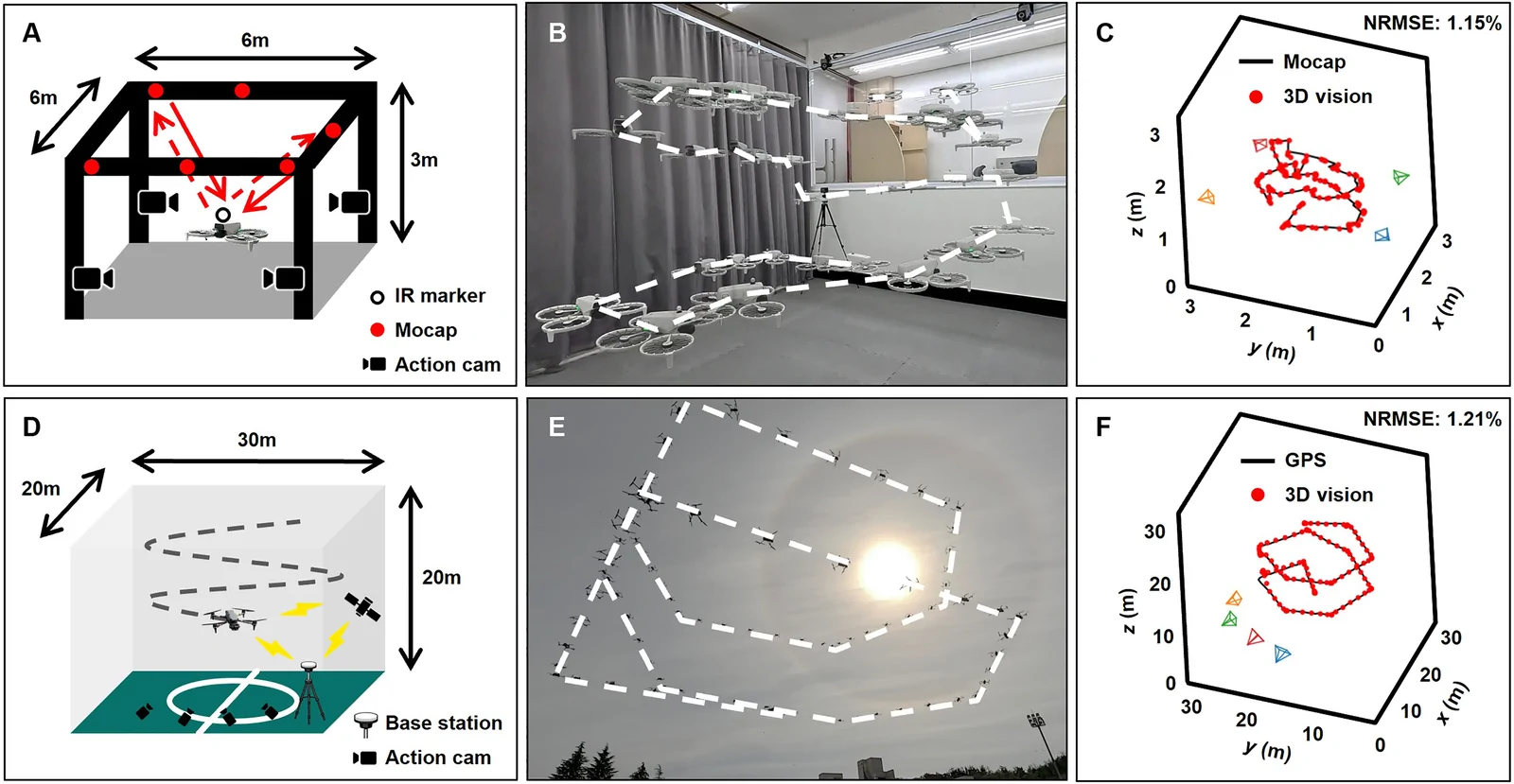
Indoor and outdoor validation of the DAM3T multicamera calibration. (**A**) Schematic of the indoor validation experiment using a motion-capture camera system (mocap) as GT within a 6 m–by–6 m–by–3 m measurement volume. IR markers mounted on the drone enable simultaneous tracking by the mocap system, while the drone is observed by the DAM3T cameras. (**B**) Superimposed image showing the drone executing a spiral trajectory within the indoor measurement volume. (**C**) Comparison between the 3D trajectory reconstructed by DAM3T (red) and the reference trajectory measured by the mocap system (black), showing close agreement (NRMSE = 1.15%). (**D**) Schematic of the outdoor validation experiment using RTK-GPS as the reference measurement in a ∼30 m–by–20 m–by–20 m field environment. (**E**) Superimposed outdoor camera image showing the drone flying a spiral trajectory across the measurement volume. (**F**) Comparison between the DAM3T-reconstructed trajectory (red) and the RTK-GPS trajectory (black), demonstrating accurate large-scale outdoor reconstruction (NRMSE = 1.21%).

Indoor and outdoor GT datasets were used to quantify the trajectory accuracy of DAM3T. For indoor validation, experiments were conducted within a 6 m–by–6 m–by–3 m measurement volume instrumented with an optical motion-capture system consisting of six Halo S13 cameras, which provided GT trajectory measurements (Fig. 2A). Spiral and tilted-eight figure flights were performed within the volume and visualized by overlaying drone images from a fixed camera view (Fig. 2B and movie S1 for the spiral flight; fig. S1D and movie S2 for the tilted-eight figure flight). After aligning the reconstructed DAM3T trajectory with the motion-capture trajectory using a similarity transform (s,R,t), where s is the isotropic scale factor, R is the 3D rotation matrix, and t is the translation vector, the spiral flight produced a normalized root-mean-square error (NRMSE) of 1.15% (RMSE = 0.0228 m; Fig. 2C). The tilted-eight figure trajectory yielded an NRMSE of 1.72% (RMSE = 0.0332 m; fig. S1E). Component-wise comparisons of the reconstructed and GT positions further confirm close agreement across all axes (fig. S1, B and F). These results establish baseline reconstruction accuracy under controlled indoor conditions with minimal environmental disturbances.

Outdoor validation was performed in a larger measurement volume of ∼20 m by 30 m by 20 m using RTK-GPS as the GT reference (fig. S1A) (*27*). A rover receiver mounted on a DJI Matrice 4T and a D-RTK 3 base station provided the RTK-GPS measurements (Fig. 2D). Spiral and tilted-eight figure flights were again executed and visualized by overlaying drone images from a fixed camera view (Fig. 2E and movie S3 for the spiral flight; fig. S1G,and movie S4 for the tilted-eight figure flight). Similarly, aligning the DAM3T trajectory with the RTK-GPS trajectory using a similarity transform resulted in NRMSEs of 1.21% for the spiral flight (RMSE = 0.236 m; Fig. 2F) and 0.40% for the tilted-eight figure flight (RMSE = 0.074 m; fig. S1H). Component-wise trajectory comparisons further demonstrate strong agreement between DAM3T and RTK-GPS measurements (fig. S1, C and I). The outdoor experiments exhibit slightly lower normalized errors than the indoor tests. One plausible explanation is the apparent target size in the image; in the outdoor setup, the drone occupies fewer pixels, which reduces the sensitivity of centroid-based localization to pixel-level boundary noise. To evaluate calibration generalization, a fixed similarity transform estimated from one trajectory was applied to the other trajectory without reestimation. Applying the transform obtained from the spiral flight to the tilted-eight figure trajectory resulted in an NRMSE of 0.55% (RMSE = 0.101 m), whereas applying the transform estimated from the tilted-eight figure trajectory to the spiral flight yielded an NRMSE of 1.22% (RMSE = 0.236 m). These results indicate that calibration derived from a single trajectory can generalize reasonably well across flight patterns. Having established the accuracy of the DAM3T reconstruction, the framework is next applied to Lagrangian flow measurements using passive tracers to demonstrate its capability for outdoor fluid-dynamics experiments.

Additional tests using two and three cameras were conducted to assess the robustness of DAM3T under reduced camera configurations. For the spiral trajectory, the NRMSEs were 1.21% (RMSE = 0.235 m; fig. S2A) and 1.16% (RMSE = 0.226 m; fig. S2B) using two and three cameras, respectively. For the tilted-eight figure trajectory, the NRMSEs were 0.49% (RMSE = 0.091 m; fig. S2D) and 0.46% (RMSE = 0.085 m; fig. S2E), respectively. To evaluate the generalization of the estimated transform, the transform obtained from the spiral trajectory was applied to reconstruct the tilted-eight figure trajectory, resulting in NRMSEs of 0.80 and 0.68% using two and three cameras, respectively (fig. S2C). Conversely, the transform obtained from the tilted-eight figure trajectory reconstructed the spiral trajectory with an NRMSE of 1.77% for both camera configurations (fig. S2F). These results indicate that DAM3T maintains stable georeferenced reconstruction even when only two cameras are used.

### Passive tracer tracking

**Fig. 3.**
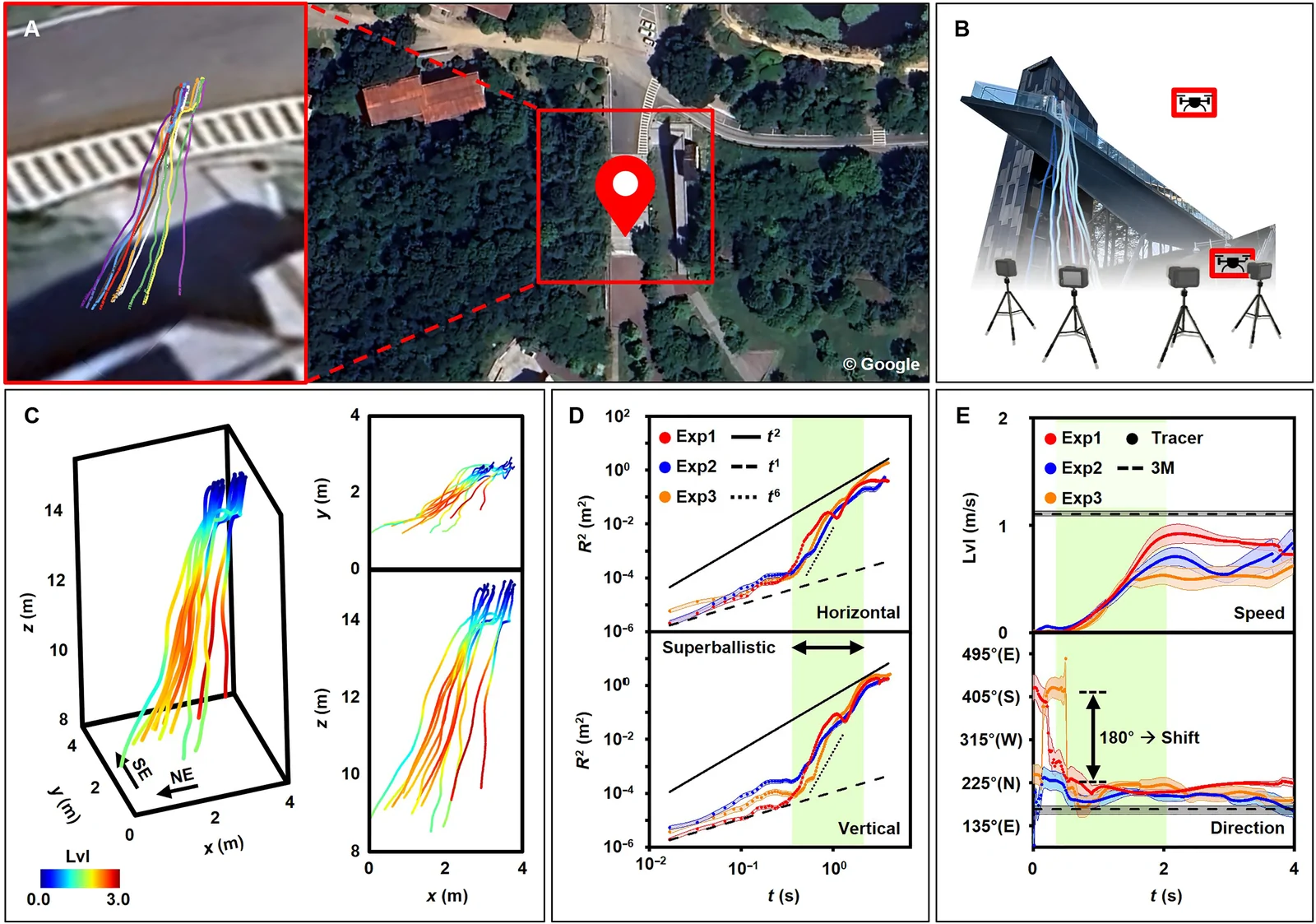
Passive tracer tracking for outdoor flow measurements. (**A**) Georeferenced visualization of the 3D trajectories on a real-world map. (**B**) Outdoor experimental setup for passive tracer tracking using the DAM3T multicamera system. (**C**) Reconstructed tracer trajectories visualized in three dimensions, with additional x−y and x−z projections illustrating the flow-driven particle motion. (**D**) Horizontal and vertical pair-dispersion statistics computed from tracer trajectories of three independent experiments, revealing the temporal evolution of particle separation in the flow. (**E**) Time series of tracer velocity magnitude and direction in the x−y plane. The reconstructed motion is compared with reference wind measurements obtained from a drone operating near ground level.

Passive tracer tracking was conducted for Lagrangian analysis of near-tower flow by releasing LED balloon tracers from the top platform of a 15-m tower, with the measurement site visualized in Google Earth using a KML file (Fig. 3, A and B). Tracer aerodynamic properties were characterized separately using indoor free-fall experiments (note S1, fig. S9, and table S1). The reconstructed 3D trajectories (Fig. 3C; fig. S3, A and B; and movies S5 to S7) are used to compute pair dispersion, a classical diagnostic in Lagrangian turbulence analysis that quantifies the temporal evolution of separation between tracer pairs (*28*). For a tracer pair (i,j), the separation distance is defined as $r_{\mathrm{ij}}\left(t\right)=\left\|x_{i}\left(t\right)-x_{j}\left(t\right)\right\|$, where $x_{i}\left(t\right)$ denotes the position of tracer i at time t. Pair dispersion is then defined as the mean-squared change in separation relative to the initial time $t_{0}$, $R^{2}\left(t\right)={\left\langle {\left[r_{\mathrm{ij}}\left(t\right)-r_{\mathrm{ij}}\left(t_{0}\right)\right]}^{2}\right\rangle }_{i<j}$. Near the tower, tracer pairs initially separate through random fluctuations before the tracers fully respond to the background wind. Because of the large tracer size and high Stokes number, the particle response time exceeds the characteristic timescale of the flow. As a result, the early-time dispersion exhibits a diffusive regime, characterized by approximately linear growth with time (Fig. 3D) (*29*). As the tracers accelerate under the influence of the background wind, the dispersion enters a superballistic regime, where the measured scaling follows $R^{2}\left(t\right)\propto t^{6}$, consistent with behavior reported in a kinematic-simulation study of pair dispersion (*30*). Beyond this interval, dispersion transitions to a ballistic regime, where $R^{2}\left(t\right)$ scales approximately as $t^{2}$, reflecting advection-dominated separation under a more coherent background flow (*31*). This sequence of regimes indicates a gradual transition from inertia-dominated particle motion to mean-flow-driven transport.

The horizontal reference wind measured by drones is compared with the horizontal transport speed derived from the tracer trajectories (Fig. 3E). At each time t, the horizontal velocity is averaged over all simultaneously observed tracers to obtain the mean horizontal speed ${\bar{v}}_{h}\left(t\right)$ and the mean direction. The tracer motion direction rapidly aligns with the reference wind direction during the transition from the superballistic to ballistic regime. One primary reason why ${\bar{v}}_{h}\left(t\right)$ remains lower than the drone-measured reference wind speed is tracer inertia (*32*). This effect can be quantified using the Stokes number, defined as $\mathrm{St}\equiv \tau_{p}/\tau_{f}$, where $\tau_{p}$ is the tracer relaxation time, and $\tau_{f}$ is the characteristic timescale of the background flow. Under the experimental conditions, the resulting value St≈23.8 indicates that the tracers respond slowly to changes in the background wind. This large Stokes number leads to a delayed adjustment of tracer velocity to the ambient flow, producing a systematic discrepancy between the reference wind speed and the tracer-derived transport speed. Passive flying sensors with substantially smaller inertia, such as wind-dispersed seed–inspired passive fliers (*33*–*36*), could reduce this discrepancy, achieving low terminal velocity and rapid aerodynamic response to ambient flows. Georeferenced reconstruction of tracer transport further allows the measured trajectories to be integrated with terrain, surrounding structures, and local meteorological observations, supporting field-scale analysis of atmospheric flow.

### Active object identification

**Fig. 4.**
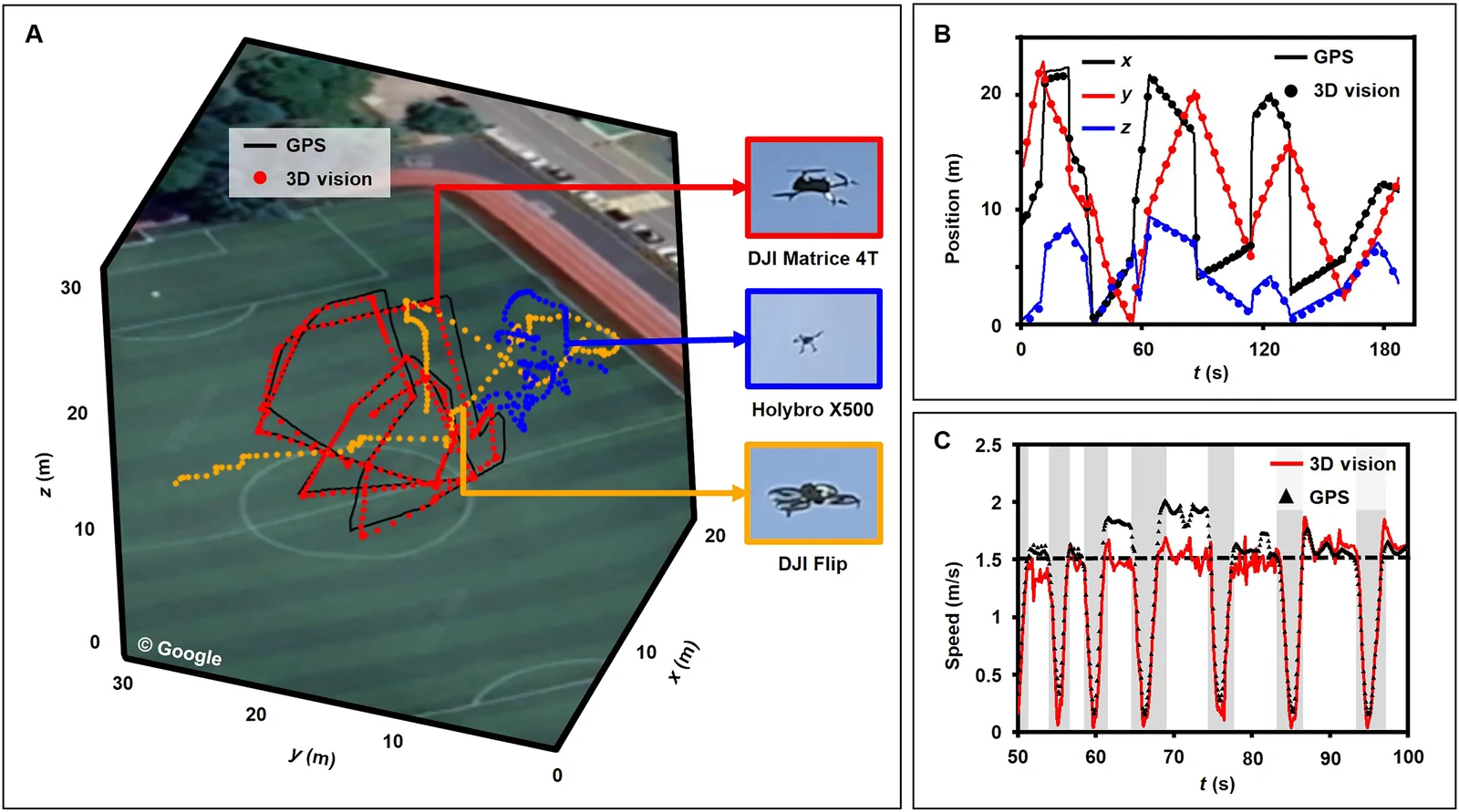
Active object identification and trajectory reconstruction. (**A**) Reconstructed 3D trajectories of three different drones identified and tracked by DAM3T. (**B**) Time series of the reconstructed *x*, *y*, and *z* coordinates of the DJI Matrice 4T compared with RTK-GPS GT, showing close agreement between DAM3T reconstruction and GPS measurements. (**C**) Velocity magnitude computed from the reconstructed DAM3T trajectory and RTK-GPS trajectory, further confirming accurate dynamic motion reconstruction.

Active object identification demonstrates georeferenced 3D tracking for multiple UAVs within a measurement volume of ∼40 m by 30 m by 20 m. Three drones—a DJI Matrice 4T, a Holybro X500, and a DJI Flip—were simultaneously tracked using DAM3T. The reconstructed trajectories were exported in KML format and visualized in Google Earth (Fig. 4A and movies S8 and S9). For the DJI Matrice 4T, which provides RTK-GPS measurements, the DAM3T trajectory was compared against RTK-GPS GT (*13*). The resulting trajectory error corresponds to an NRMSE of 1.12% (RMSE = 0.295 m). Component-wise comparisons of the reconstructed and GT positions further confirm agreement across all axes (Fig. 4B). Similar position time series comparisons for the Holybro X500 and the DJI Flip are shown in fig. S4 (A and B).

For the DJI Matrice 4T, the corresponding speed profiles were derived from RTK-GPS and DAM3T position signals (Fig. 4C). During the experiment, the drone executed an autonomous waypoint mission with hover periods at each waypoint and a prescribed cruise speed of $v_{\text{ref}}=1.5\;m/s$ along the segments between waypoints. To evaluate steady-flight performance, the speed comparison was restricted to quasisteady segments between waypoints, excluding hover and acceleration phases. Over the selected segments, the NRMSEs relative to $v_{\text{ref}}$ equal 1.79% for DAM3T and 3.79% for RTK-GPS, indicating that DAM3T provides more consistent speed estimates under steady flight conditions. The ability to simultaneously identify and track multiple UAVs in a georeferenced coordinate system highlights the potential of DAM3T for applications such as low-altitude airspace monitoring, drone traffic management, and multiagent aerial systems research. The reconstructed trajectories are directly comparable with predefined flight corridors, geofenced areas, and restricted-zone maps. This georeferenced representation supports route-deviation assessment and safety monitoring of unexpected UAV motion.

### Sport-scene tracking

**Fig. 5.**
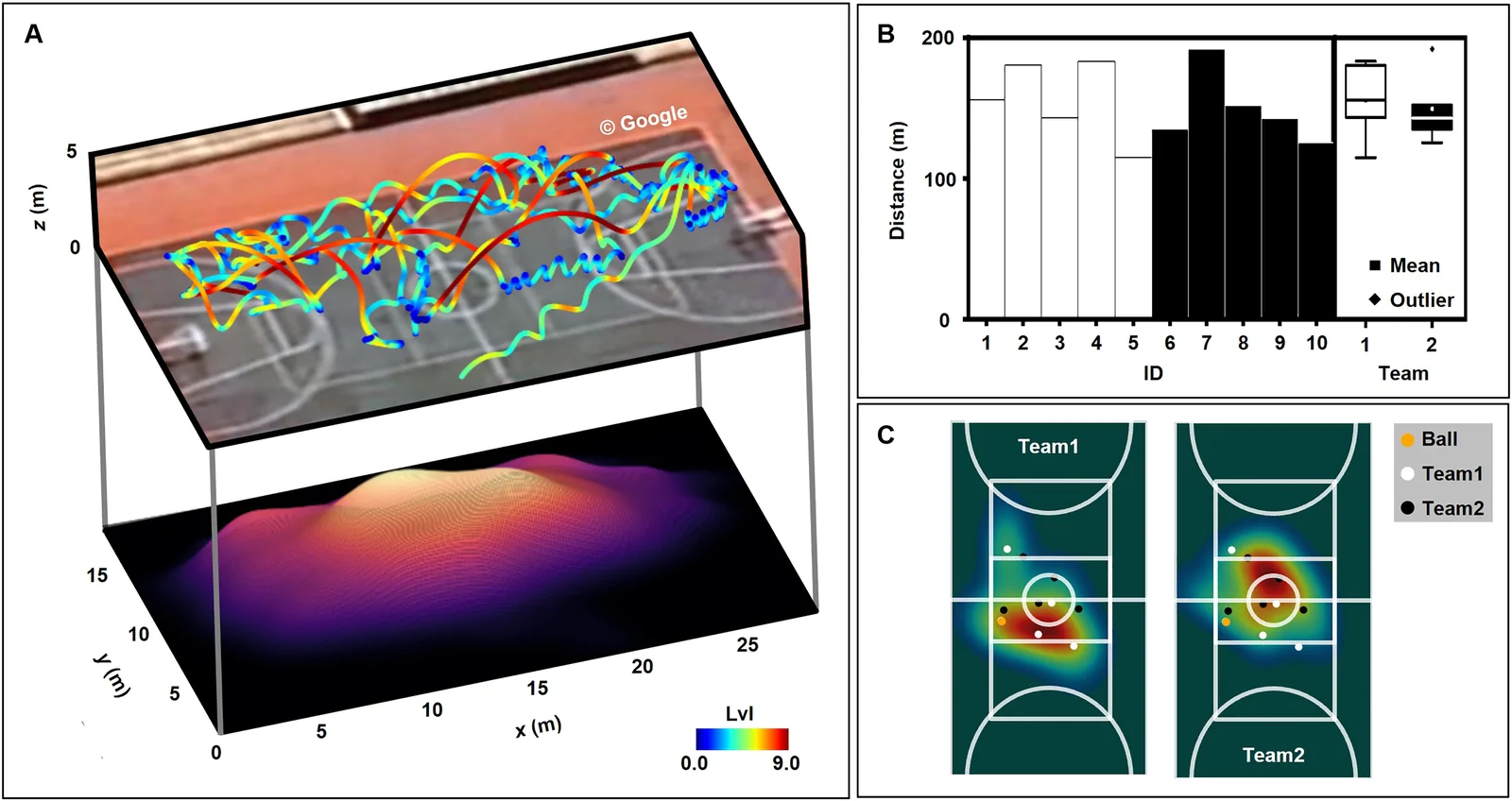
Sport-scene tracking and motion analysis. (**A**) Reconstructed 3D basketball trajectory (top) together with the players’ spatial traffic-density map (bottom) over a 116-s game segment. The density map represents the spatial distribution of player positions accumulated over time. (**B**) Player travel distances over 116 s. The left panel shows the per-player distance distribution, while the right panel summarizes per-team travel distances using a box plot. (**C**) Team spatial density maps at frame 5500 for team 1 (left) and team 2 (right), illustrating differences in spatial positioning and court coverage.

Following the demonstration of Lagrangian flow measurements using passive tracers, the versatility of DAM3T is further illustrated through multiagent motion analysis in complex outdoor scenes. Sport-scene tracking is demonstrated on a 116-s sequence of a basketball game, with 1 ball and 10 players reconstructed in 3D (movie S10). Figure 5A (top) shows the reconstructed ball trajectory, where instantaneous velocity magnitude is computed from frame-to-frame displacement and encoded using a color map. Distinct motion patterns corresponding to dribbling and passing can be identified from the trajectory geometry. Dribble segments appear as repeated short parabolic arcs characterized by low speed (∣v∣≤4 m/s), low height (z≤1 m), and horizontal travel distance below 1 m. In contrast, pass segments appear as single long arcs with larger speeds, greater height, and horizontal travel distances exceeding 4 m. Figure 5A (bottom) visualizes a player traffic-density map derived from the tracked player positions (*37*). The court is divided into a spatial grid, and the player dwell time is accumulated within each cell. The resulting density field highlights spatial usage of the court, with the highest traffic concentrated near the center and gradually decreasing toward the boundaries.

Player workload is further quantified using the total traveled distance over the same 116-s interval (Fig. 5B). The traveled distance reflects spatial coverage and role-dependent movement during the game segment. Team-level distributions are summarized using box plots. The mean traveled distances are 155.4 m for team 1 and 149.4 m for team 2, corresponding to a difference of 6.0 m. The distribution for team 1 exhibits a larger interquartile range, indicating greater variability in player activity levels. In contrast, team 2 shows a more compact distribution, although one player appears as an outlier relative to the remaining team members. Figure 5C visualizes the team-wise spatial distribution at frame 5500 using a density map (*38*). The player density is distributed using an elliptical kernel aligned with each player’s motion direction, with intensity scaled according to speed. This representation highlights instantaneous movement flow and directional occupancy across the court. Team-specific density fields enable a direct comparison of ally and opponent concentrations, while the ball position provides additional context for formation-level interpretation and supports qualitative identification of candidate passing options and movement corridors (*39*). This shared spatial reference enables court-based sports analysis by relating player and ball motion to physical court geometry while supporting noncontact, multitarget tracking without wearable sensors.

Together, these results demonstrate that DAM3T enables portable and scalable 3D measurements across diverse outdoor environments. By combining drone-assisted calibration with synchronized multicamera tracking, the framework supports georeferenced trajectory reconstruction for aerial vehicles, passive tracers, and multiagent motion analysis. This capability opens opportunities for quantitative measurements of complex dynamic systems in field applications such as aircraft monitoring, atmospheric fluid mechanics, and human crowd dynamics, where traditional laboratory measurement techniques are difficult to deploy.

## DISCUSSION

This study presents DAM3T as a field-deployable system for quantitative measurement of 3D trajectories of moving targets in outdoor environments. By using an unmodified drone as a calibration target, DAM3T estimates multicamera extrinsic parameters across large observation volumes without requiring dedicated calibration markers. Alignment of the reconstructed trajectories with RTK-GPS measurements further enables georeferenced tracking in the global coordinate system (WGS84), integrating multicamera calibration and geographic alignment into a single automated workflow. Validation against indoor motion-capture and outdoor RTK-GPS measurements confirms the accuracy of the proposed calibration framework. Notably, DAM3T maintained low RMSE values with only two to four cameras, demonstrating robust georeferenced 3D tracking with compact camera configurations (fig. S5). Beyond calibration, the results demonstrate the versatility of DAM3T across multiple outdoor measurement scenarios. The system enables Lagrangian flow analysis using LED tracers, tracking of multiple UAVs, and multiagent motion analysis in sports scenes, where reconstructed trajectories can be related to real-world spatial references such as flow regions, flight paths, terrain, or field geometry. In particular, the passive-tracer experiments highlight the potential of DAM3T for outdoor Lagrangian flow studies. The large Stokes number of the current LED balloon tracers (St≫1) indicates that their response to background flow remains limited, suggesting that future experiments will benefit from passive sensors with substantially lower inertia. Recent developments in bioinspired microfliers and floaters, such as wind-dispersed seed–inspired passive flying sensors, provide a promising direction for achieving this capability (*35*).

Several practical challenges remain in large-scale field measurements, including background noise, illumination variation, partial occlusion, and appearance similarity among targets during multiobject tracking. These factors can influence the stability of 2D observations and the continuity of reconstructed trajectories. Increasing view overlap, either by incorporating aerial viewpoints or by expanding the number of cameras, offers a promising strategy for reducing occlusion and improving tracking robustness in complex outdoor environments. DAM3T can further be extended from 3D position tracking to six-degree-of-freedom motion analysis, broadening its applicability to dynamic systems involving coupled translational and rotational motion (fig. S6 and movie S17). Because the framework uses the drone itself as the calibration target, the approach is not limited to camera-based systems and can naturally extend to cross-modal calibration with other sensing modalities such as LiDAR. This extensibility highlights the potential of DAM3T as a foundation for scalable sensor-fusion infrastructure for studying complex dynamic systems in natural environments.

## MATERIALS AND METHODS

### Hardware setup

The DAM3T system consisted of ground-mounted cameras and a calibration drone (Fig. 1F). The modular architecture enabled operation with as few as two cameras, while additional cameras expanded the measurement volume and improved reconstruction accuracy. In the current implementation, up to four GoPro HERO12 cameras were arranged to surround the investigation area. For georeferenced measurements, a DJI Matrice 4T drone equipped with an RTK-GPS receiver provided GT data.

### Time synchronization

Two time-synchronization strategies were used depending on the experimental setting. Most experiments used the GoPro Timecode function, in which timecode metadata were assigned to each camera by scanning a QR code generated in the GoPro Quik application. The official GoPro specification reported a synchronization error below 50 ms. The effective residual delay was further evaluated using a 100-Hz counter display, and its influence on 3D reconstruction accuracy was analyzed (note S2 and fig. S10). For a representative target speed of 10 m/s, a viewing-ray intersection angle of 45°, and an angle of 45° between the target velocity direction and the viewing ray of the delayed camera, the predicted synchronization-induced error was ∼0.1 m. In a subset of experiments, audio-based synchronization in Adobe Premiere Pro was also used. Under short-baseline and low-noise conditions, a slate signal was used to facilitate alignment. Both approaches avoided dedicated wireless synchronization devices and hardware trigger units.

### Multicamera calibration

Intrinsic parameters and lens distortion coefficients were estimated before the experiments at the target recording resolution of 4 K (*40*). At least 20 checkerboard images with a 7×4 corner pattern were captured at diverse viewing angles and distances to achieve uniform coverage of the image plane. For multicamera calibration, the drone calibration trajectory and camera placement were jointly designed to keep the drone target within the overlapping observation volume while providing broad image-plane coverage and depth variation from each camera viewpoint (fig. S8, A and C). The detected 2D calibration points were distributed across each camera image plane rather than being confined to a limited region (fig. S8, B and D). This depth variation improved geometric conditioning for triangulation and increases the stability of the estimated extrinsic parameters. The RTK-GPS trajectory served as GT and enabled the alignment of camera and target coordinates with the world frame in WGS84. Figure 1G summarized the overall workflow.

### Multiview 2D trajectories

The drone flew within the overlapping field of view of the cameras while following either a spiral trajectory or a tilted-eight figure trajectory. Depending on the scene and tracking reliability, either a multiple-object tracking (MOT) pipeline or a single-object tracking (SOT) pipeline generated the 2D trajectory in each camera view. For the MOT pipeline, a YOLOv11x-based (*41*) detector trained using the publicly available Drone-Detection-YOLOv11x repository (*42*) detected the drone, and ByteTrack (*43*) linked the detections across frames. Geometric filtering suppressed spurious background detections. Lucas-Kanade optical flow further stabilized the bounding box by combining motion-based predictions with detection results and then applying exponential smoothing. Background tracks caused by static jitter were removed, and valid anchor tracks were identified. Compatible anchor segments were connected, and missing trajectory segments were recovered through interpolation and smoothing. For the SOT pipeline, the OpenCV Channel and Spatial Reliability Tracker (CSRT) (*44*) tracked the drone. The bounding box was initialized in the first frame of each sequence, and CSRT updated the target position frame by frame. When drift or target loss occurred, the bounding box was reinitialized. For both pipelines, the final 2D trajectory in each view was represented by the center coordinates of the bounding box at each frame.

MOT was adopted when it produced reliable trajectories without reinitialization, whereas SOT was used otherwise. The frame-wise matching rate was defined as the percentage of frames in which the distance between the MOT and SOT bounding-box centers was below 100 px at a 4K resolution. MOT was applied to the outdoor validation (fig. S7, C and D) and the active object identification (fig. S7F), yielding a mean matching rate of 96.46% relative to the corresponding SOT results. SOT was used for the indoor validation (fig. S7, A and B), passive tracer tracking (fig. S7E), and sport-scene tracking (fig. S7G); the average agreement between MOT and SOT across these applications was 63.02%.

### Essential matrix estimation

For camera view c and frame t, the 2D observation $u_{c,t}\in {\mathbb{R}}^{2}$ was undistorted and normalized using the intrinsic matrix and distortion parameters $\left(K_{c},\theta_{c}\right)$ to obtain the normalized image point $x_{c,t}=\pi [K_{c}^{-1}\text{undist}\left(u_{c,t},\theta_{c}\right)\text{]}$, where $x_{c,t}={\left[x,y,1\right]}^{⊤}$. Two views (i,j) were selected, and the essential matrix E was estimated from correspondences $x_{i,t}↔x_{j,t}$ using the random sample consensus algorithm (*45*). The estimated essential matrix satisfied the epipolar constraint, $x_{j,t}^{⊤}Ex_{i,t}=0$, and its decomposition, E=[t]×R, yielded the relative pose (R,t) (*46*). Triangulation then reconstructed an initial 3D drone trajectory $\left\{X_{t}\right\}$ from the selected two-view correspondences. For each remaining view c, the camera pose was estimated from 3D-2D correspondences $X_{t}↔u_{c,t}$ using a Perspective-n-Point solver with the random sample consensus, yielding the extrinsic parameters of the remaining cameras. For each frame t, multiview triangulation reconstructed the 3D position from observations from at least two cameras, producing the final 3D trajectory.

### GPS-based georeferencing

A similarity transform (s,R,t) aligned the DAM3T trajectory $\left\{p_{t}\right\}$ to the RTK-GPS GT trajectory $\left\{g_{t}\right\}$, with the transformed DAM3T point defined as ${\hat{g}}_{t}=\mathrm{sR}p_{t}+t$, where s>0 and R∈SO (3). To improve alignment stability, the drone remained stationary for at least 5 s at the beginning and the end of the flight. This stationary period provided a common temporal segment in both trajectories. The trajectories were then resampled along arc length to match the number of points. The Umeyama algorithm (*47*) provided an initial estimate of (s,R,t), and an L-BFGS-B optimizer (*48*) refined the parameters by solving the RMSE minimization problem $\underset{s,R,t}{\text{min}}\frac{1}{N}\sum_{t=1}^{N}{\left\|g_{t}-\left(\mathrm{sR}p_{t}+t\right)\right\|}_{2}^{2}$. The iterative closest point algorithm (*49*) then updated the transform on the original trajectories to obtain the final (s,R,t). Applying the estimated transform to the DAM3T local coordinates converted the reconstructed positions to WGS84 coordinates.

### Error metric

The trajectory error was reported using the NRMSE. Given the aligned trajectory ${\left\{{\hat{g}}_{t}\right\}}_{t=1}^{N}$ and the GT trajectory ${\left\{g_{t}\right\}}_{t=1}^{N}$, the RMSE distance was defined as $\text{RMSE}=\sqrt{\frac{1}{N}\sum_{t=1}^{N}{\left\|{\hat{g}}_{t}-g_{t}\right\|}_{2}^{2}}$. The normalization factor was the diagonal length of the GT trajectory bounding box, $d_{\text{bb}}={\left\|\underset{t}{\text{max}}\left(g_{t}\right)\underset{t}{-\text{min}}\left(g_{t}\right)\right\|}_{2}$. The NRMSE was computed as $\text{NRMSE}\left(\%\right)=100\cdot \frac{\text{RMSE}}{d_{\text{bb}}}$.

### System validation

System validation used two GT configurations. In the motion-capture setup, cameras with integrated infrared (IR) laser illumination recorded signals reflected from a retroreflective marker attached to the target (Fig. 2A). An IR filter suppressed visible background signals and increased the contrast of the reflected marker signal. Hardware synchronization enabled simultaneous multicamera observations across cameras, and multiview triangulation reconstructed the 3D position. Four GoPro HERO12 cameras were placed at the corners of the measurement volume, and a DJI Flip carrying the IR marker was manually flown along spiral and tilted-eight figure trajectories. According to the manufacturer, the motion-capture system had a nominal accuracy of 0.4 mm.

In the outdoor RTK-GPS setup, a base station was installed at a fixed location and streamed real-time differential corrections to a rover receiver to reduce common-mode GPS errors (Fig. 2D). The system reliably received signals from more than 30 satellites across multiple constellations and provided nominal accuracies of 0.8 cm in the horizontal direction and 1.5 cm in the vertical direction. Four GoPro HERO12 cameras were arranged in an approximately linear configuration, and a DJI Matrice 4T followed predefined spiral and tilted-eight figure trajectories under autonomous control.

### Passive tracer tracking

A total of 11 or 12 LED balloons were released three times from the Haedong Tower observatory at POSTECH, ∼15 m above ground level. Wind data were recorded simultaneously using two hovering drones positioned at least 5 m from the release point. A DJI Matrice 4T hovered near the release altitude (15 m) sampling at 10 Hz, and a DJI Mavic 3M hovered near ground-level (5 m) sampling at 5 Hz. Four cameras were arranged in a fan-shaped configuration to cover the balloon release region, and drone-assisted calibration using a tilted-eight figure trajectory yielded an NRMSE of 1.83% (RMSE = 0.268 m; fig. S8E and movie S11). In each camera view, CSRT generated 2D tracer trajectories. Multiview triangulation then reconstructed 3D Lagrangian trajectories from these observations, followed by outlier rejection. In this process, DAM3T calibration linked the reconstructed tracer motion to the global coordinate system (WGS84), allowing tracer transport to be interpreted relative to the release site and surrounding structures. The velocity was obtained by time differentiation of the reconstructed positions. A Kalman filter and a rolling mean filter were used to suppress noise.

### Active object identification

A DJI Matrice 4T followed a predefined route in autonomous flight, while a Holybro X500 and a DJI Flip were flown manually within the measurement volume. Four cameras were arranged in a fan-shaped configuration to cover the UAV measurement volume, and DAM3T calibration using a tilted-eight figure trajectory yielded an NRMSE of 0.65% (RMSE = 0.120 m; fig. S8F and movie S12). CSRT estimated the drone position in each camera view on a frame-by-frame basis, and the resulting 2D trajectories were reconstructed into 3D trajectories. Multicamera calibration provided the geometric parameters required to align DAM3T coordinates with WGS84 and obtain georeferenced 3D paths. The velocity was computed by differentiating the estimated positions with respect to time. To reduce noise amplification, outliers were removed using the median absolute deviation criterion, and missing samples were filled by linear interpolation. A rolling median filter followed by a rolling mean filter was applied to smooth the resulting signals. Because the velocity time series included stop-and-go behavior resulting from the waypoint mission profile, quasisteady segments were selected by masking monotonic speed ramps with ∆v≥0.5 m/s, which corresponded to acceleration or deceleration near waypoints. Samples with v≤0.3 m/s were removed to reduce noise dominance near zero speed. NRMSE was then computed on the remaining segments using $v_{\text{ref}}$ as the reference.

### Sport-scene tracking

Three cameras were placed around the outdoor basketball court to provide multiview coverage of the players and ball, and multicamera calibration using a tilted-eight figure trajectory yielded an NRMSE of 1.39% (RMSE = 0.305 m; fig. S8G and movie S13). CSRT estimated the ball position in each camera view on a frame-by-frame basis. Player trajectories were obtained by detecting 10 players using YOLOv8 and applying BoT-SORT for multiobject tracking and reidentification. Multiview triangulation reconstructed the resulting 2D trajectories into 3D trajectories. Here, multicamera calibration placed the reconstructed player and ball motion within a georeferenced coordinate system, enabling the trajectories to be related to the physical court geometry. After outlier removal and interpolation, the Kalman filter reduced residual noise. All participants provided written informed consent before data collection.

## Supplementary Materials

### The PDF file includes:

Supplementary Notes S1 and S2 Figs. S1 to S10 Table S1 Legends for movies S1 to S17 Legend for DAM3T Manual References

### Other Supplementary Material for this manuscript includes the following:

Movies S1 to S17

DAM3T Manual
