# Supplementary material for: Large-scale outdoor 3D trajectory measurements enabled by drone-assisted multicamera tracking
Source: Sci Adv. 2026 Aug 19;12(34):eaeh8352. doi: 10.1126/sciadv.aeh8352 (PMC13488927; doi:10.1126/sciadv.aeh8352)
Supplement: Supplementary file 1 [file sciadv.aeh8352_sm.pdf]

Science Advances
AAAS

Supplementary Materials for

Large-scale outdoor 3D trajectory measurements enabled by drone-assisted multicamera tracking

Jungyeon Kim *et al.*

Corresponding author: Jin-Tae Kim, jimmy516@postech.ac.kr

Sci. Adv. **12**, eaeh8352 (2026)
DOI: 10.1126/sciadv.aeh8352

The PDF file includes:

Supplementary Notes S1 and S2
Figs. S1 to S10
Table S1
Legends for movies S1 to S17
Legend for DAM3T Manual
References

Other Supplementary Material for this manuscript includes the following:

Movies S1 to S17
DAM3T Manual

Supplementary Note

Supplementary Note S1. Passive tracer characterization

Prior to outdoor flow measurements, the tracer dynamics were characterized in an indoor environment without wind disturbances*(5)*. Three helium-filled LED balloons were released in free-fall, and each balloon was tested in three repeated drops (Table S1). A tripod-mounted Sony A7S III camera was positioned horizontally to record the motion, and the balloon positions were extracted using CSRT tracking (Fig. S9A). The vertical position was differentiated with respect to time to estimate the falling velocity, and the terminal velocity was measured as $v_{\mathrm{t}} = 1.731 \pm 0.1129\,\mathrm{m/s}$ (Fig. S9B; Table S1; Movies S14-S16).

The drag coefficient was estimated from the force balance at terminal velocity. Assuming dynamic equilibrium between drag and the apparent weight of the tracer W_{app}, the drag force satisfies $F_{\mathrm{D}} \approx W_{\mathrm{app}} = F_{\mathrm{g}} - F_{\mathrm{b}} = mg - \rho V g$, where m is the tracer mass, V is the tracer volume, $\rho = 1.225\,\mathrm{kg/m^3}$ is the air density, and g is the gravitational acceleration. The drag force is expressed as $F_{\mathrm{D}} = \frac{1}{2}\rho A v_{\mathrm{t}}^2 C_{\mathrm{D}}$, where A is the frontal area and C_{D} is the drag coefficient. The three balloons used in the free-fall experiment had a major-axis length of 20 cm and an average minor-axis length of 17.54 cm, giving an estimated frontal area range of $A = 241.63 - 275.52\,\mathrm{cm^2}$. The drag coefficient range was estimated using the measured frontal-area variation among the racers. The apparent tracer weight was obtained from the scale measurements as $W_{\mathrm{app}} = 2.1\,\mathrm{g} \times g$. Using the measured ranges of frontal area and terminal velocity, the drag coefficient was estimated as $C_{\mathrm{D}} = 0.438 \pm 0.056$, consistent with the literature*(50)*.

Supplementary Note S2. Effect of time delay on 3D reconstruction accuracy

The residual synchronization error of the GoPro cameras was first evaluated using a 100 Hz counter display. The counter value changed every 10 ms and was simultaneously recorded by four GoPro cameras under different frame-rate settings of 60, 120, and 240 fps (Fig. S6A). Across these settings, the maximum counter discrepancy among the four cameras was one counter step, corresponding to a residual relative time delay of approximately 10 ms.

The influence of this residual time delay on 3D reconstruction accuracy was then analyzed. For a target moving with velocity v, a relative delay Δt introduced an apparent positional mismatch of $\Delta s = v\Delta t$. Only the component of this displacement perpendicular to the viewing ray contributes to image-plane mismatch. Therefore, for camera i, the effective mismatch was approximated as $\Delta s_{\text{eff},i} \approx \|v\|\Delta t \sin\alpha_i$, where α_i denotes the angle between the target velocity vector and the viewing ray of the delayed camera. The resulting triangulation error can therefore be approximated as $E_{3D} \approx \frac{\Delta s_{\text{eff},i}}{\sin\theta}$, where θ was defined as the intersection angle between the viewing rays. Thus, the error per unit target speed was given by $\frac{E_{3D}}{\|\mathbf{v}\|} \approx \frac{\Delta t \sin\alpha_i}{\sin\theta}$. Under the measured delay condition of $\Delta t \approx 10\ ms$, the predicted error increased when the target motion was more perpendicular to the delayed camera viewing ray and when the viewing-ray intersection angle decreased (Fig. S6B).

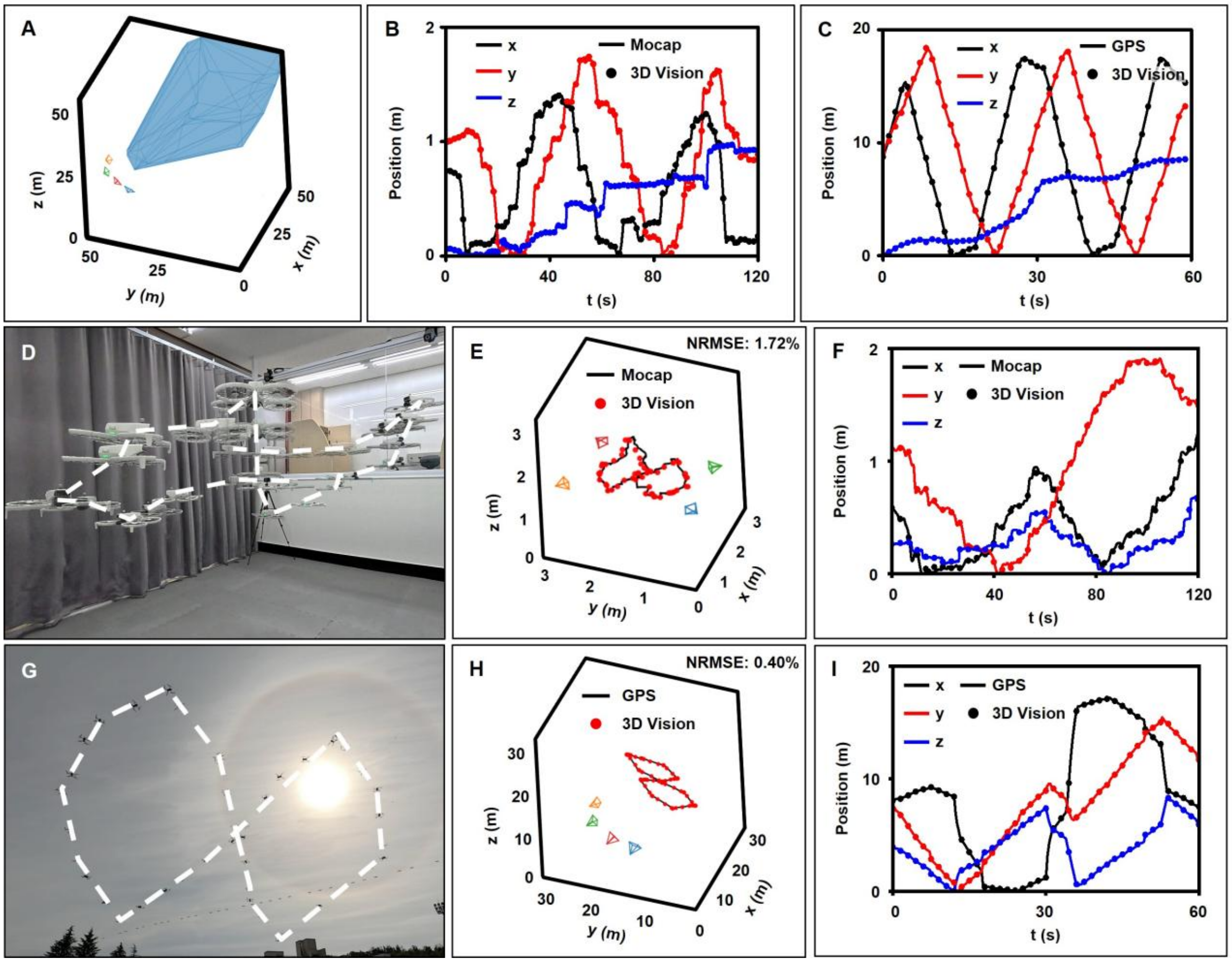

Fig. S. 1 | **A** Investigation area of the outdoor measurement volume. **B** Time series of the x-, y-, and z-coordinates of DJI Flip along the spiral trajectory, comparing DAM3T with mocap ground truth. **C** Time series of the x-, y-, and z-coordinates of DJI Matrice 4T during the spiral trajectory, with DAM3T results and RTK-GPS ground truth. **D** Superimposed image of the drone executing a tilted-eight figure trajectory within the indoor measurement volume. **E** Reconstructed 3D trajectory from DAM3T (red) and the reference trajectory measured by the mocap system (black), indicating close agreement (NRMSE = 1.72 %). **F** Time series of the x-, y-, and z-coordinates of DJI Flip compared with mocap ground truth. **G** Superimposed image showing the drone flying a tilted-eight figure across the outdoor measurement volume. **H** The DAM3T-reconstructed trajectory (red) and the RTK-GPS trajectory (black), demonstrating accurate large-scale outdoor reconstruction (NRMSE = 0.40 %). **I** Time series of the x-, y-, and z-coordinates of DJI Matrice 4T, with DAM3T results and RTK-GPS ground truth.

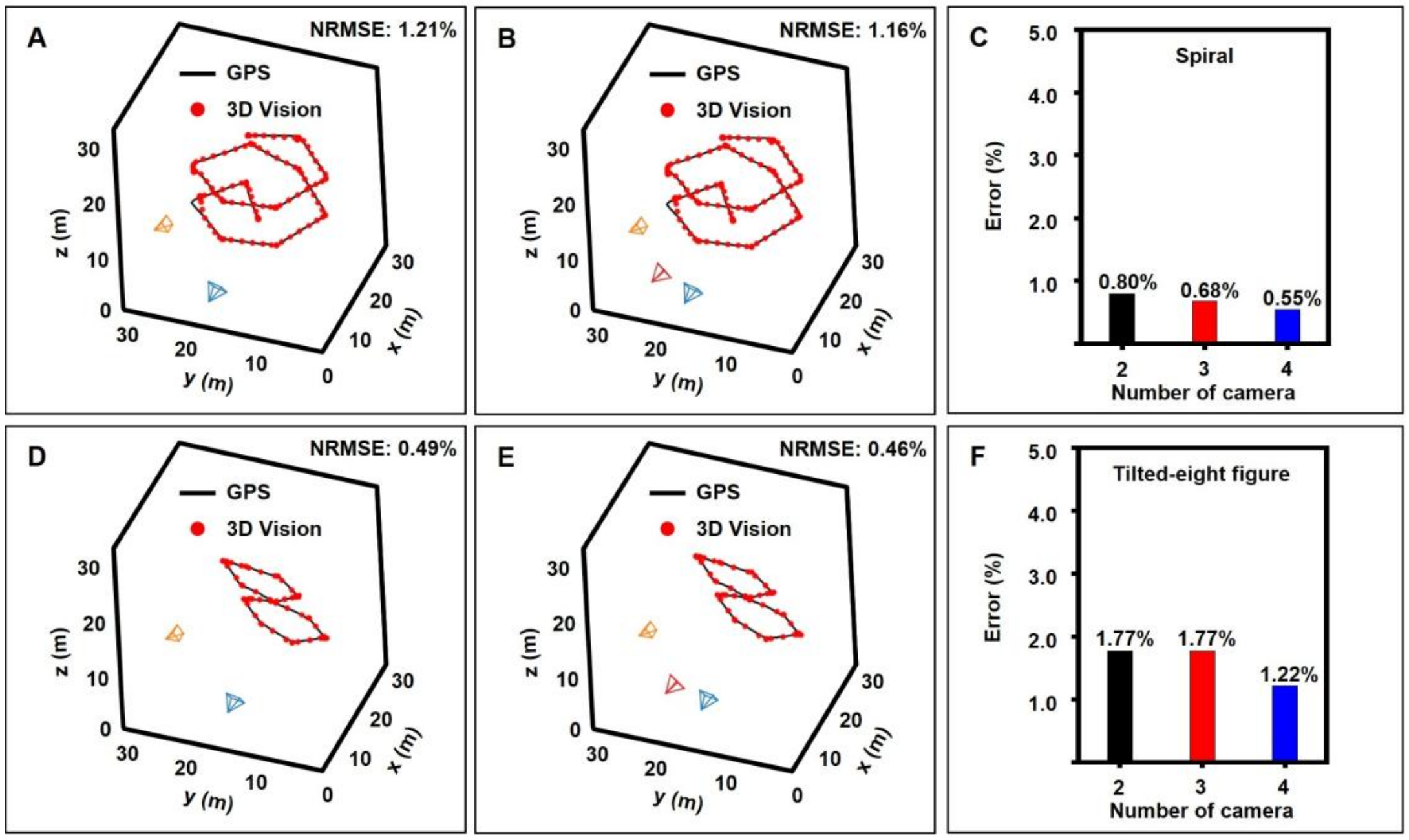

Fig. S. 2 | Effect of camera number on outdoor validation. **A-B** Comparison between the DAM3T-reconstructed spiral trajectory (red) and the RTK-GPS trajectory (black) using two and three cameras, respectively (NRMSE = 1.21 % and 1.16 %). **C** NRMSEs for the tilted-eight figure trajectory using two, three, and four cameras, including results using the transform estimated from the spiral trajectory. **D-E** Comparison between the DAM3T-reconstructed tilted-eight figure trajectory (red) and the RTK-GPS trajectory (black) using two and three cameras, respectively (NRMSE = 0.49 % and 0.46 %). **F** NRMSEs for the spiral trajectory using two, three, and four cameras, including results using the transform estimated from the tilted-eight figure trajectory.

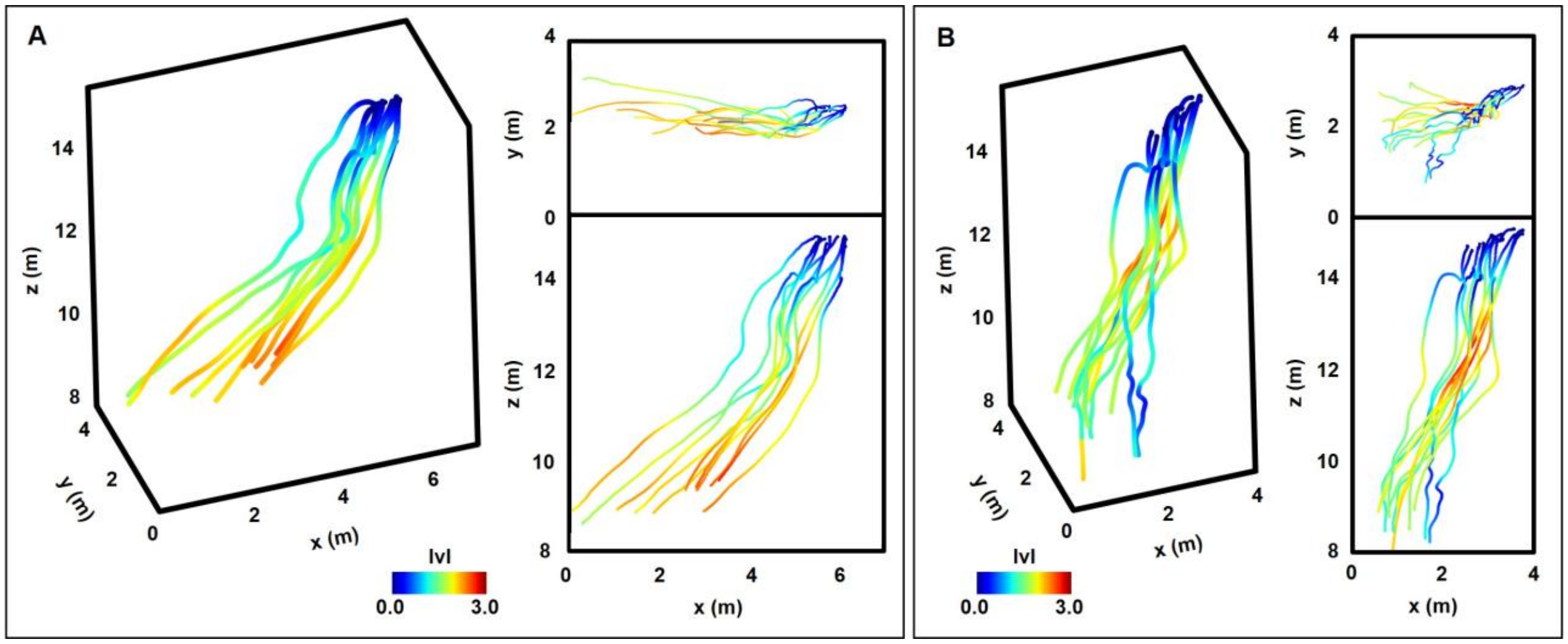

Fig. S. 3 | **A-B** Reconstructed tracer trajectories from two separate experiments visualized in three dimensions, with additional $x-y$ and $x-z$ projections illustrating the flow-driven particle motion.

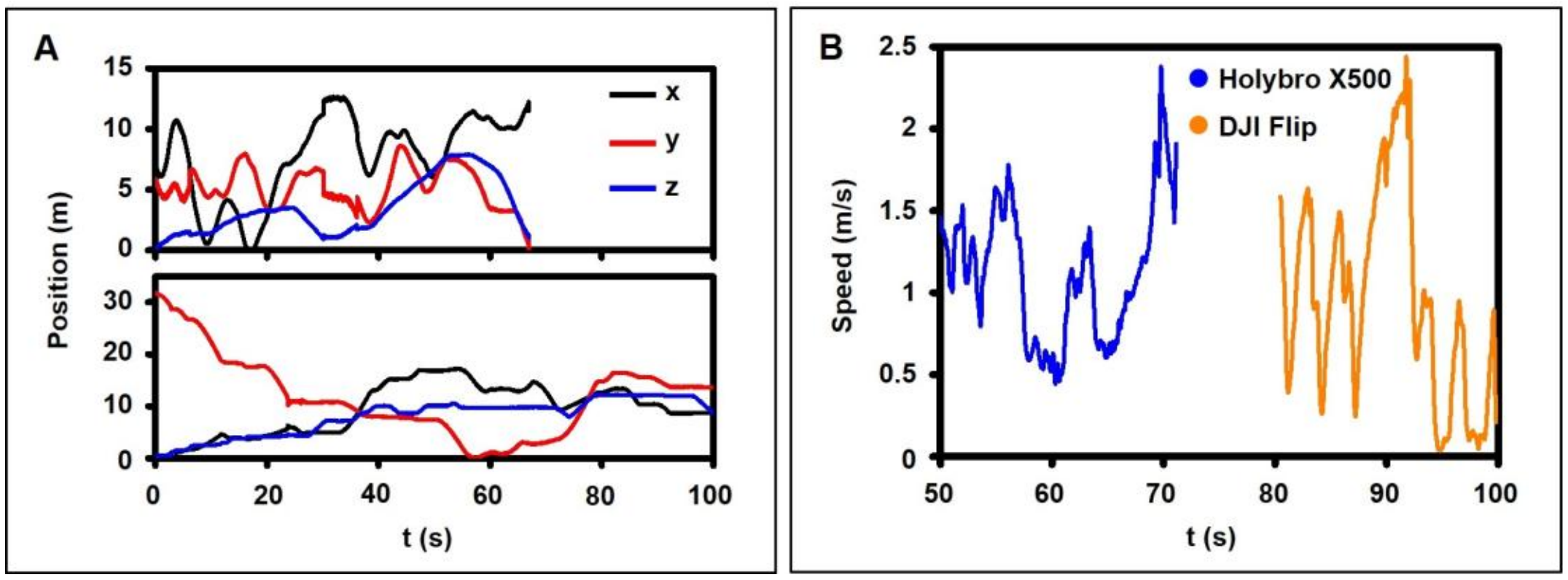

Fig. S. 4 | A Time series of the x-, y-, and z-coordinates of Holybro X500 and DJI Flip reconstructed by DAM3T. **B** Speed magnitude computed from the reconstructed DAM3T trajectories.

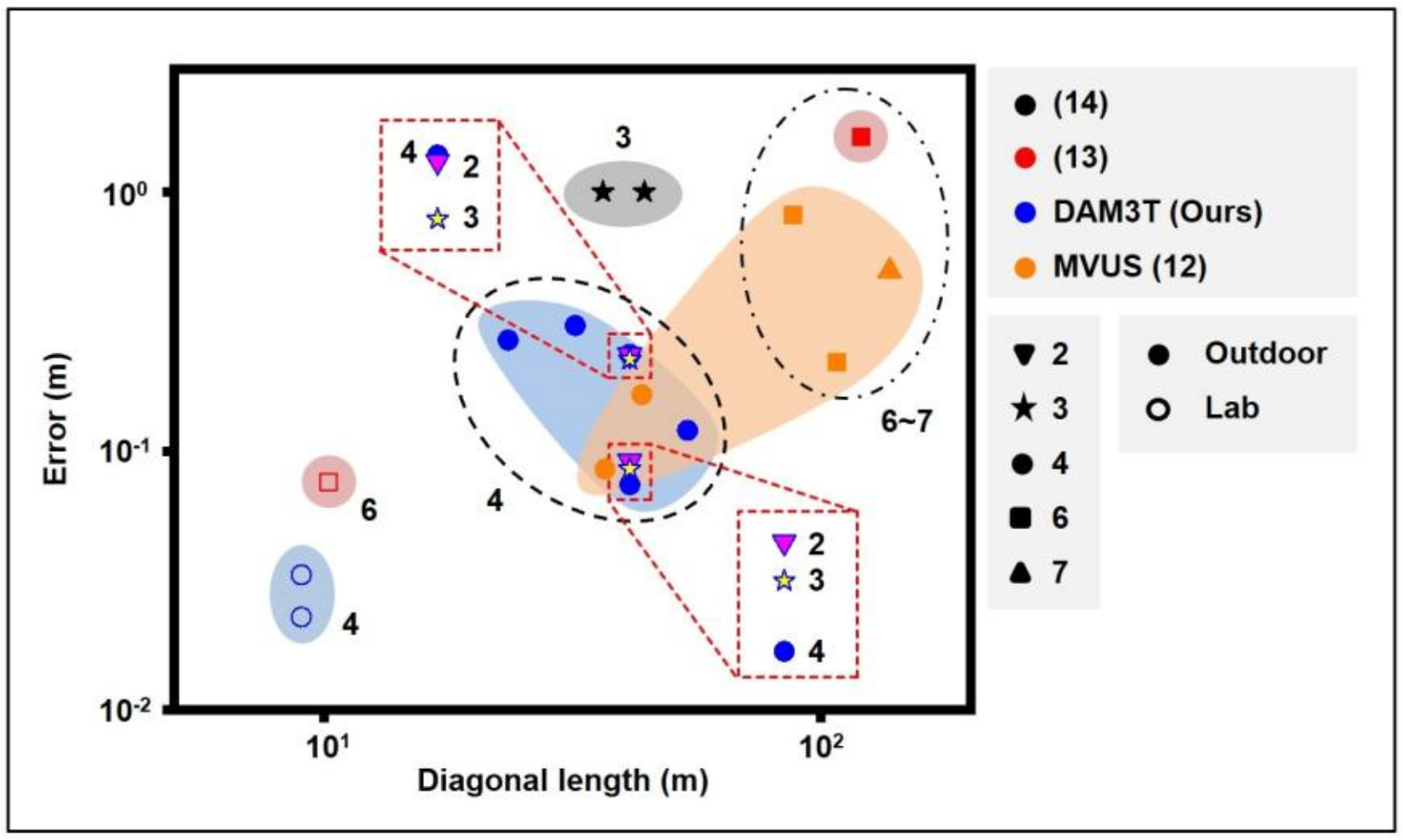

Fig. S. 5 | Position error comparison with measurement scale.

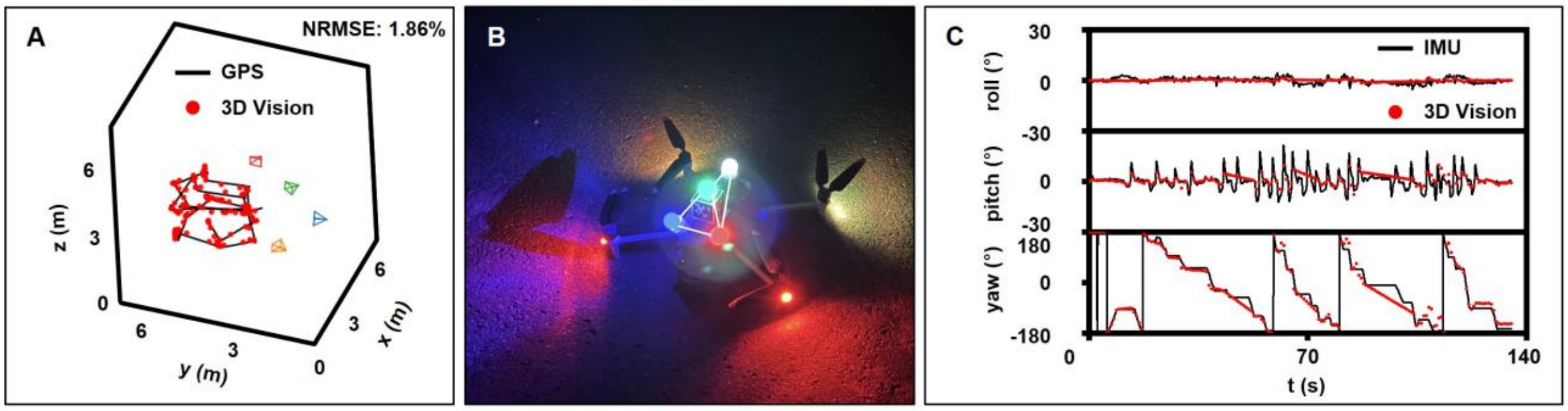

Fig. S. 6 | A Calibration trajectory used for the 6-DoF demonstration, showing the DAM3T-reconstructed trajectory (red) and RTK-GPS trajectory (black) (NRMSE = 1.86 %; RMSE = 0.0815 m). **B** Four non-coplanar LED markers attached to the drone. **C** Estimated roll, pitch, and yaw angles compared with the drone onboard IMU-based attitude estimates.

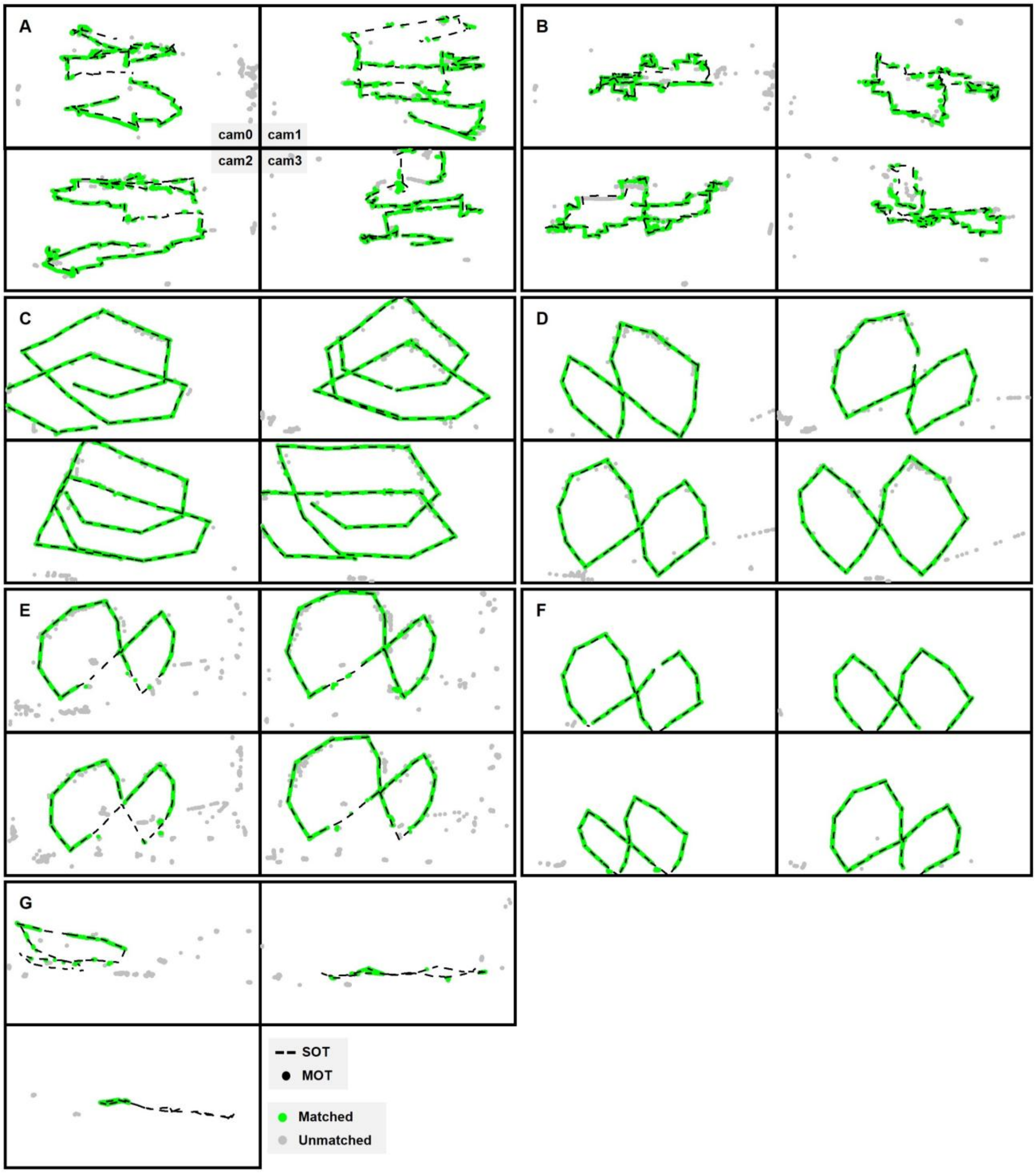

Fig. S. 7 | Frame-level matching points using a single object tracker (SOT) and a multiple object tracker (MOT). **A-B** Spiral and tilted-eight figure trajectories in indoor validation. **C-D** Spiral and tilted-eight figure trajectories in outdoor validation. **E** Tilted-eight figure trajectory in passive tracer tracking. **F** Tilted-eight figure trajectory in active object identification. **G** Tilted-eight figure trajectory in sport-scene tracking.

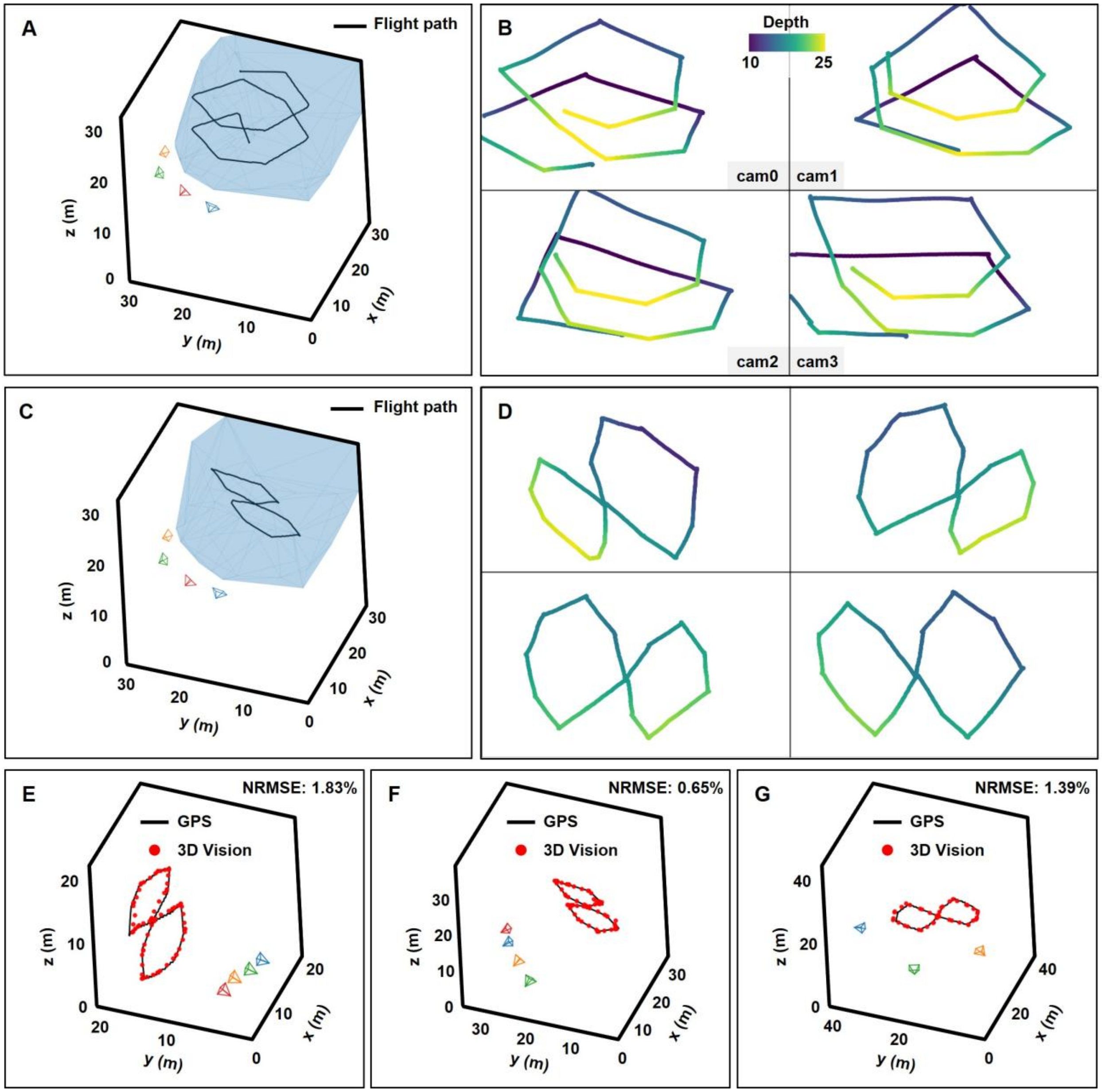

Fig. S. 8 | A Investigation area of the outdoor measurement volume with the spiral trajectory. **B** Image-plane distribution of detected 2D calibration points for the spiral trajectory. **C** Investigation area of the outdoor measurement volume with the tilted-eight figure trajectory. **D** Image-plane distribution of detected 2D calibration points for the tilted-eight figure trajectory. **E-G** Calibration trajectory reconstruction in representative outdoor experiments. (**E**) DAM3T-reconstructed calibration trajectory (red) and RTK-GPS trajectory (black) for passive tracer tracking (NRMSE = 1.83 %). (**F**) DAM3T-reconstructed calibration trajectory (red) and RTK-GPS trajectory (black) for active object identification (NRMSE = 0.65 %). (**G**) DAM3T-reconstructed calibration trajectory (red) and RTK-GPS trajectory (black) for sport-scene analysis (NRMSE = 1.39 %).

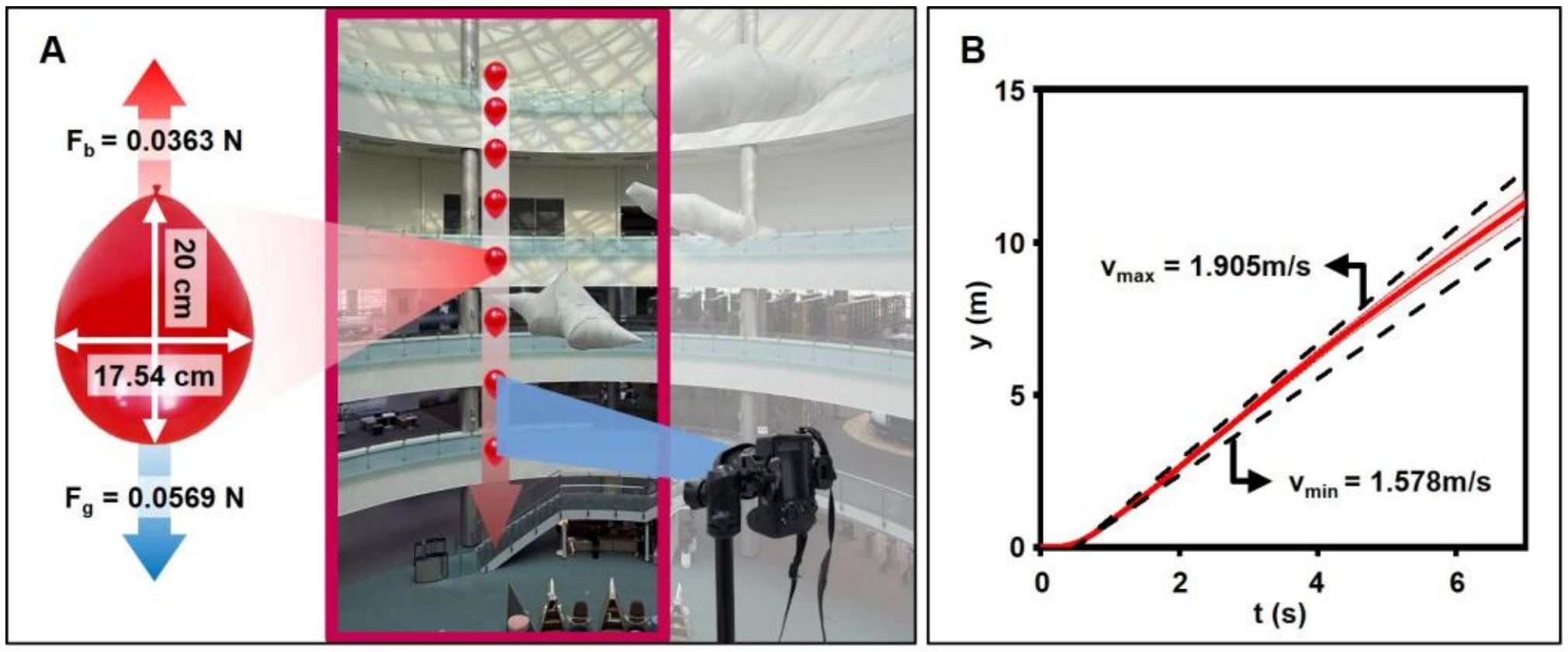

Fig. S9. | **A** Still-air free-fall experiment used to characterize the tracer dynamics and verify force balance during decent. (left) free-body diagram of the helium balloon tracer, including buoyance F_{b} and gravitational force F_{g}. **B** Vertical displacement of the tracer during free-fall as a function of time, with $y = 0$ defined at the release height and positive y directed downward. The measured velocity range indicates near-constant descent speed.

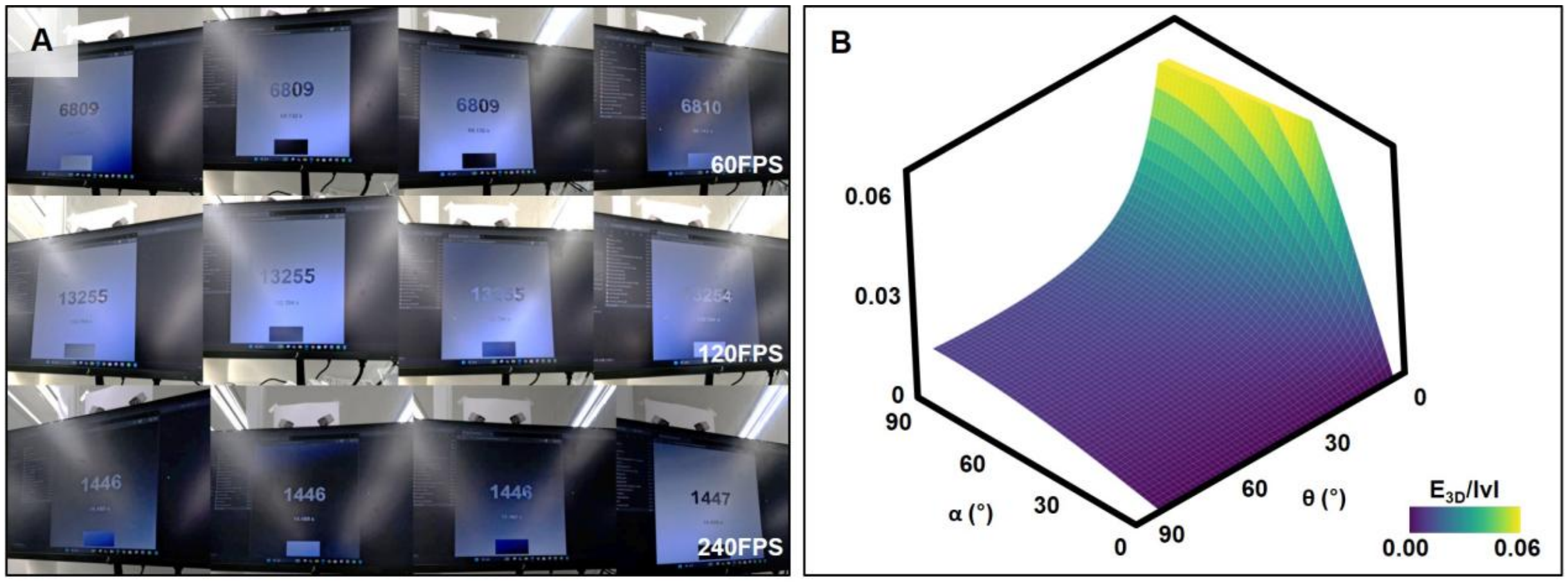

Fig. S. 10 | A Counter display test used to estimate the residual synchronization delay among four GoPro cameras at 60, 120, and 240 fps. **B** Predicted 3D reconstruction error per unit target speed under a 10 ms delay as a function of target-motion angle and viewing-ray intersection angle.

	Red			Blue			White			Average
v_t (m/s)	1.598	1.905	1.685	1.708	1.884	1.700	1.578	1.728	1.789	1.731

Table S1. | Free-fall test results of LED balloon tracers.

Movie. S1. | Indoor validation experiment of spiral trajectory, showing time-synchronized 2D tracking results and the corresponding 3D reconstruction at 6x playback speed.

Movie. S2. | Indoor validation experiment of tilted-eight figure trajectory, showing time-synchronized 2D tracking results and the corresponding 3D reconstruction at 6x playback speed.

Movie. S3. | Outdoor validation experiment of spiral trajectory, showing time-synchronized 2D tracking results and the corresponding 3D reconstruction at 6x playback speed.

Movie. S4. | Outdoor validation experiment of tilted-eight figure trajectory, showing time-synchronized 2D tracking results and the corresponding 3D reconstruction at 6x playback speed.

Movie. S5. | Passive tracer tracking experiment 1, showing time-synchronized 2D tracking results and 3D reconstructed trajectories with speed magnitude colored by speed magnitude.

Movie. S6. | Passive tracer tracking experiment 2, showing time-synchronized 2D tracking results and 3D reconstructed trajectories with speed magnitude colored by speed magnitude.

Movie. S7. | Passive tracer tracking experiment 3, showing time-synchronized 2D tracking results and 3D reconstructed trajectories with speed magnitude colored by speed magnitude.

Movie. S8. | Active object identification showing time-synchronized 2D tracking results of DJI Matrice 4T and Holybro X500, together with the corresponding 3D reconstruction at 2x playback speed.

Movie. S9. | Active object identification showing time-synchronized 2D tracking results of DJI Matrice 4T and DJI Flip, together with the corresponding 3D reconstruction at 2x playback speed.

Movie. S10. | Sport-scene tracking showing time-synchronized 2D tracking results and 3D reconstruction of the ball and players, with the ball trajectory colored by speed magnitude.

Movie. S11. | Passive tracer tracking calibration experiment of tilted-eight figure trajectory, showing time-synchronized 2D tracking results and the corresponding 3D reconstruction at 6x playback speed.

Movie. S12. | Active object identification calibration experiment of tilted-eight figure trajectory, showing time-synchronized 2D tracking results and the corresponding 3D reconstruction at 6x playback speed.

Movie. S13. | Sport-scene analysis calibration experiment of tilted-eight figure trajectory, showing time-synchronized 2D tracking results and the corresponding 3D reconstruction at 6x playback speed.

Movie. S14. | Free-fall test of a red LED balloon tracer, showing CSRT-based tracking of its vertical motion in three repeated trials.

Movie. S15. | Free-fall test of a blue LED balloon tracer, showing CSRT-based tracking of its vertical motion in three repeated trials.

Movie. S16. | Free-fall test of a white LED balloon tracer, showing CSRT-based tracking of its vertical motion in three repeated trials.

Movie. S17. | 6-DoF motion tracking demonstration, showing time-synchronized 2D tracking results of four non-coplanar LED markers attached to a drone and the corresponding 3D reconstruction at 6x playback speed.

DAM3T Manual | Manual for the DAM3T data and code repository

REFERENCES

1. J. Li, M. Guala, J. Hong, Field investigation of 3-D snow settling dynamics under weak atmospheric turbulence. *J. Fluid Mech.* **997**, A33 (2024).

2. N. Bristow, J. Li, P. Hartford, M. Guala, J. Hong, Imaging-based 3D particle tracking system for field characterization of particle dynamics in atmospheric flows. *Exp. Fluids* **64**, 78 (2023).

3. Y. Xiang, T. Zhang, C. Ma, L. Lv, J. Liu, W. Liu, Y. Cheng, Lidar vertical observation network and data assimilation reveal key processes driving the 3-D dynamic evolution of PM 2.5 concentrations over the North China Plain. *Atmos. Chem. Phys.* **21**, 7023–7037 (2021).

4. F. Köpp, S. Rahm, I. Smalikho, Characterization of aircraft wake vortices by 2-μ m pulsed Doppler lidar. *J. Atmos. Oceanic Tech.* **21**, 194–206 (2004).

5. J. Gonzalo, D. Domínguez, D. López, J. Fernández, Lighter-than-air particle velocimetry for wind speed profile measurement. *Renew. Sustain. Energy Rev.* **33**, 323–332 (2014).

6. N. Krishnakumar, S. Sharma, S. K. Pal, J. Hong, 3D characterization of smoke plume dispersion using multi-view drone swarm. *Sci. Total Environ.* **980**, 179466 (2025).

7. S. L. Goodrick, G. L. Achtemeier, N. K. Larkin, Y. Liu, T. M. Strand, Modelling smoke transport from wildland fires: A review. *Int. J. Wildland Fire* **22**, 83–94 (2013).

8. M. Roeleke, U. E. Schlägel, C. Gallagher, J. Pufelski, T. Blohm, R. Nathan, S. Toledo, F. Jeltsch, C. C. Voigt, Insectivorous bats form mobile sensory networks to optimize prey localization: The case of the common noctule bat. *Pro. Natl. Acad. Sci. U.S.A.* **119**, e2203663119 (2022).

9. L. Jin, M. Ghirardelli, J. Mann, M. Sjöholm, S. T. Kral, J. Reuder, Rotary-wing drone-induced flow—Comparison of simulations with lidar measurements. *Atmos. Meas. Tech.* **17**, 2721–2737 (2024).

10. T. A. Abir, E. Kuantama, R. Han, J. Dawes, R. Mildren, P. Nguyen, “Towards robust Lidar-based 3D detection and tracking of UAVs,” in *Proceedings of the Ninth Workshop on Micro Aerial Vehicle Networks, Systems, and Applications* (ACM, 2023), pp. 1–7.

11. D. H. Theriault, N. W. Fuller, B. E. Jackson, E. Bluhm, D. Evangelista, Z. Wu, M. Betke, T. L. Hedrick, A protocol and calibration method for accurate multi-camera field videography. *J. Exp. Biol.* **217**, 1843–1848 (2014).

12. J. Li, J. Murray, D. Ismaili, K. Schindler, C. Albl, "Reconstruction of 3D flight trajectories from ad-hoc camera networks," in *2020 IEEE/RSJ International Conference on Intelligent Robots and Systems (IROS)* (IEEE, 2020), pp. 1621–1628.

13. A. Rozantsev, S. N. Sinha, D. Dey, P. Fua, "Flight dynamics-based recovery of a UAV trajectory using ground cameras," in *2017 IEEE Conference on Computer Vision and Pattern Recognition (CVPR)* (IEEE, 2017), pp. 6030–6039.

14. N. J. Sie, S. Srigrarom, S. Huang, "Field test validations of vision-based multi-camera multi-drone tracking and 3D localizing with concurrent camera pose estimation," in *2021 6th International Conference on Control and Robotics Engineering (ICCRE)* (IEEE, 2021), pp. 139–144.

15. S. Srigrarom, N. J. L. Sie, H. Cheng, K. H. Chew, M. Lee, P. Ratsamee, "Multi-camera multi-drone detection, tracking and localization with trajectory-based re-identification," in *2021 Second International Symposium on Instrumentation, Control, Artificial Intelligence, and Robotics (ICA-SYMP)* (IEEE, 2021), pp. 1–6.

16. D. Commun, C. Pradalier, M. Balchanos, O. Fischer, D. Mavris, "Evaluation of a drone-based camera calibration approach for hard-to-reach cameras," in *2021 IEEE International Conference on Robotics and Automation (ICRA)* (IEEE, 2021), pp. 13149–13155.

17. W. Feng, Z. Su, Y. Han, H. Liu, Q. Yu, S. Liu, D. Zhang, Inertial measurement unit aided extrinsic parameters calibration for stereo vision systems. *Opt. Lasers Eng.* **134**, 106252 (2020).

18. F. Bottalico, C. Niezrecki, A. Sabato, Preliminary validation of a hybrid visual-inertial stereocamera calibration for 3D point tracking using independent UAVs. *Measurement* **252**, 120287 (2026).

19. L. Shi, X. Lan, X. Lan, T. Zhang, Quick calibration of massive urban outdoor surveillance cameras. *ISPRS J. Photogramm. Remote Sens.* **214**, 231–243 (2024).

20. X. Lan, L. Zhou, B. Lin, J. Li, G. Lv, Rapid survey method for large-scale outdoor surveillance cameras using binary space partitioning. *ISPRS J. Photogramm. Remote Sens.* **207**, 57–73 (2024).

21. S. Zhuge, X. Xu, Y. He, B. Lin, S. Gan, X. Zhang, 3-D motion trajectory measurement for the target through a multi-UAVs system. *Measurement* **204**, 112088 (2022).

22. G. Pandey, J. McBride, S. Savarese, R. Eustice, "Automatic targetless extrinsic calibration of a 3D lidar and camera by maximizing mutual information," in *Proceedings of the Twenty-Sixth AAAI Conference on Artificial Intelligence* (AAAI Press, 2012), pp. 2053–2059.

23. S. Miyata, H. Saito, K. Takahashi, D. Mikami, M. Isogawa, H. Kimata, "Ball 3D trajectory reconstruction without preliminary temporal and geometrical camera calibration," in *Proceedings of the IEEE Conference on Computer Vision and Pattern Recognition Workshops (CVPRW)* (IEEE, 2017), pp. 164–169.

24. J. Kim, M. Ra, H. Lee, J. Kim, W.-Y. Kim, Precise 3D baseball pitching trajectory estimation using multiple unsynchronized cameras. *IEEE Access* **7**, 166463–166475 (2019).

25. P. Wu, Y. Li, D. Xue, UAV target tracking: A survey. *Artif. Intell. Rev.* **58**, 358 (2025).

26. W. Wu, M. Xu, Q. Liang, L. Mei, Y. Peng, Multi-camera 3D ball tracking framework for sports video. *IET Image Process.* **14**, 3751–3761 (2020).

27. Z. Niu, H. Xia, P. Tao, T. Ke, Accuracy assessment of UAV photogrammetry system with RTK measurements for direct georeferencing. *ISPRS Ann. Photogramm. Remote Sens. Spatial Inf. Sci.* **X-1-2024**, 169–176 (2024).

28. L. F. Richardson, Atmospheric diffusion shown on a distance-neighbour graph. *Proc. R. Soc. A* **110**, 709–737 (1926).

29. J. T. Kim, J. Nam, S. Shen, C. Lee, L. P. Chamorro, On the dynamics of air bubbles in Rayleigh-Benard convection. *J. Fluid Mech.* **891**, A7 (2020).

30. D. Thomson, B. Devenish, Particle pair separation in kinematic simulations. *J. Fluid Mech.* **526**, 277–302 (2005).

31. G. K. Batchelor, Diffusion in a field of homogeneous turbulence: II. The relative motion of particles. *Math. Proc. Camb. Philos. Soc.* **48**, 345–362 (1952).

32. J. Zhou, H. Gao, X. Wang, J. Li, An accurate wind retrieval method based on observing hydrogen balloons with multi-theodolite measurements. *J. Atmos. Oceanic Tech.* **39**, 1297–1307 (2022).

33. B. H. Kim, K. Li, J.-T. Kim, Y. Park, H. Jang, X. Wang, Z. Xie, S. M. Won, H.-J. Yoon, G. Lee, W. J. Jang, K. H. Lee, T. S. Chung, Y. H. Jung, S. Y. Heo, Y. Lee, J. Kim, T. Cai, Y. Kim, P. Prasopsukh, Y. Yu, X. Yu, R. Avila, H. Luan, H. Song, F. Zhu, Y. Zhao, L. Chen, S. H. Han, J. Kim, S. J. Oh, H. Lee, C. H. Lee, Y. Huang, L. P. Chamorro, Y. Zhang, J. A. Rogers, Three-dimensional electronic microfliers inspired by wind-dispersed seeds. *Nature* **597**, 503–510 (2021).

34. J. T. Kim, H. J. Yoon, S. Cheng, F. Liu, S. Kang, S. Paudel, D. Cho, H. Luan, M. Lee, G. Jeong, J. Park, Y. T. Huang, S. E. Lee, M. Cho, G. Lee, M. Han, B. H. Kim, J. Yan, Y. Park, S. Jung, L. P. Chamorro, J. A. Rogers, Functional bio-inspired hybrid fliers with separated ring and leading edge vortices. *Pnas Nexus* **3**, pgae110 (2024).

35. C. Park, Z. Hu, K. Li, J. T. Kim, J. A. Rogers, Distributed multitudes of bio-inspired, biodegradable Lagrangian sensors for environmental sustainability. *Nat. Commun.* **17**, 601 (2026).

36. H. J. Yoon, G. Lee, J. T. Kim, J. Y. Yoo, H. W. Luan, S. Cheng, S. Kang, H. L. T. Huynh, H. Kim, J. Park, J. Kim, S. S. Kwak, H. Ryu, J. Kim, Y. S. Choi, H. Y. Ahn, J. Choi, S. Oh, Y. H. Jung, M. Park, W. B. Bai, Y. G. Huang, L. P. Chamorro, Y. Park, J. A. Rogers, Biodegradable,

three-dimensional colorimetric fliers for environmental monitoring. *Sci. Adv.* **8**, eade3201, (2022).

37. R. Zhang, L. Wu, Y. Yang, W. Wu, Y. Chen, M. Xu, Multi-camera multi-player tracking with deep player identification in sports video. *Pattern Recognit.* **102**, 107260 (2020).

38. B. Barron, N. Sitaraman, T. Arias, Analyzing NBA player positions and interactions with density-functional fluctuation theory. *Sci. Rep.* **15**, 19830 (2025).

39. S. Hauri, N. Djuric, V. Radosavljevic, S. Vucetic, "Multi-modal trajectory prediction of NBA players," in *2021 IEEE Winter Conference on Applications of Computer Vision (WACV)* (IEEE, 2021), pp. 1640–1649.

40. Z. Zhang, "Flexible camera calibration by viewing a plane from unknown orientations," in *Proceedings of the Seventh IEEE International Conference on Computer Vision (ICCV)* (IEEE, 1999), pp. 666–673.

41. Ultralytics, YOLO11, Ultralytics YOLO Docs (2024); https://docs.ultralytics.com/models/yolo11/ [accessed 27 March 2026].

42. D. İlmak, Drone-Detection-YOLOv11x, GitHub (2025); https://github.com/doguilmak/Drone-Detection-YOLOv11x [accessed 27 March 2026].

43. Y. Zhang, P. Sun, Y. Jiang, D. Yu, F. Weng, Z. Yuan, P. Luo, W. Liu, X. Wang, Bytetrack: "Multi-object tracking by associating every detection box," in *Computer Vision – ECCV 2022* (Springer, 2022), pp. 1–21.

44. A. Lukezic, T. Vojir, L. Cehovin Zajc, J. Matas, M. Kristan, "Discriminative correlation filter with channel and spatial reliability," in *2017 IEEE Conference on Computer Vision and Pattern Recognition (CVPR)* (IEEE, 2017), pp. 4847–4856.

45. M. A. Fischler, R. C. Bolles, Random sample consensus: A paradigm for model fitting with applications to image analysis and automated cartography. *Commun. ACM* **24**, 381–395 (1981).

46. R. Hartley, A. Zisserman, *Multiple View Geometry in Computer Vision* (Cambridge Univ. Press, ed. 2, 2004).

47. S. Umeyama, Least-squares estimation of transformation parameters between two point patterns. *IEEE Trans. Pattern Anal. Mach. Intell.* **13**, 376–380 (1991).

48. R. H. Byrd, P. Lu, J. Nocedal, C. Zhu, A limited memory algorithm for bound constrained optimization. *SIAM J. Sci. Comput.* **16**, 1190–1208 (1995).

49. P. J. Besl, N. D. McKay, A method for registration of 3-D shapes. *IEEE Trans. Pattern Anal. Mach. Intell.* **14**, 239–256 (1992).

50. A. Haider, O. Levenspiel, Drag coefficient and terminal velocity of spherical and nonspherical particles. *Powder Technol.* **58**, 63–70 (1989).
