# Supplementary material for: Large-scale outdoor 3D trajectory measurements enabled by drone-assisted multicamera tracking
Source: Sci Adv. 2026 Aug 19;12(34):eaeh8352. doi: 10.1126/sciadv.aeh8352 (PMC13488927; doi:10.1126/sciadv.aeh8352)
Supplement: Supplementary file 2 [file sciadv.aeh8352_dam3t_manual.zip › aeh8352_dam3t_manual.pdf]

DAM3T

Drone-Assisted Multi-camera 3D Tracking

Overview

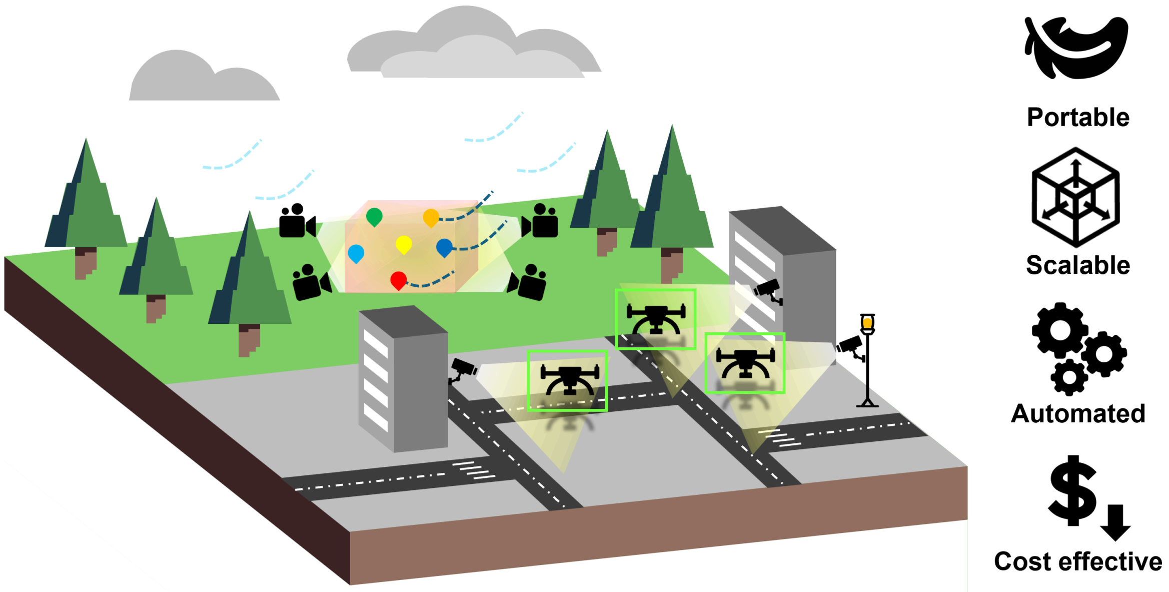

DAM3T is a field-deployable outdoor multi-camera tracking repository for 3D trajectory reconstruction in a geographic coordinates.

This repository is organized around two main workflows:

1. **DAM3T : Outdoor Validation**
   A single target is tracked in each camera view, reconstructed in 3D, and aligned to a geographic coordinate.

2. **Application : Multi-UAV Tracking**
   Multiple UAVs are triangulated in 3D using pre-estimated camera extrinsics under the same overall workflow as outdoor validation.

The repository includes utilities for:

- 2D tracking & post-processing
- Camera pose estimation & 3D Reconstruction
- GPS alignment
- Visualization and export

Pipeline Summary

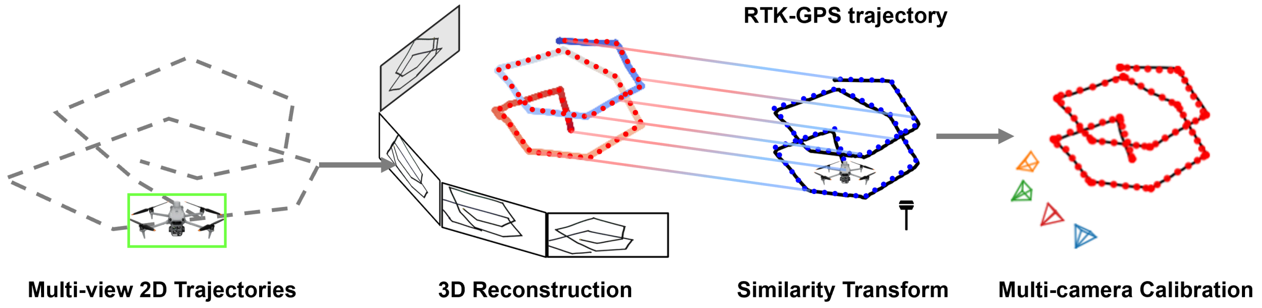

Outdoor Validation

0. In this repository, DJI flight logs are converted with '**csv_to_pkl.py**'.
1. Run `sot.py` or `mot.py` to generate 2D tracking results for each camera. `mot.py` additionally requires `mot_post.py` or `mot_post2.py`.

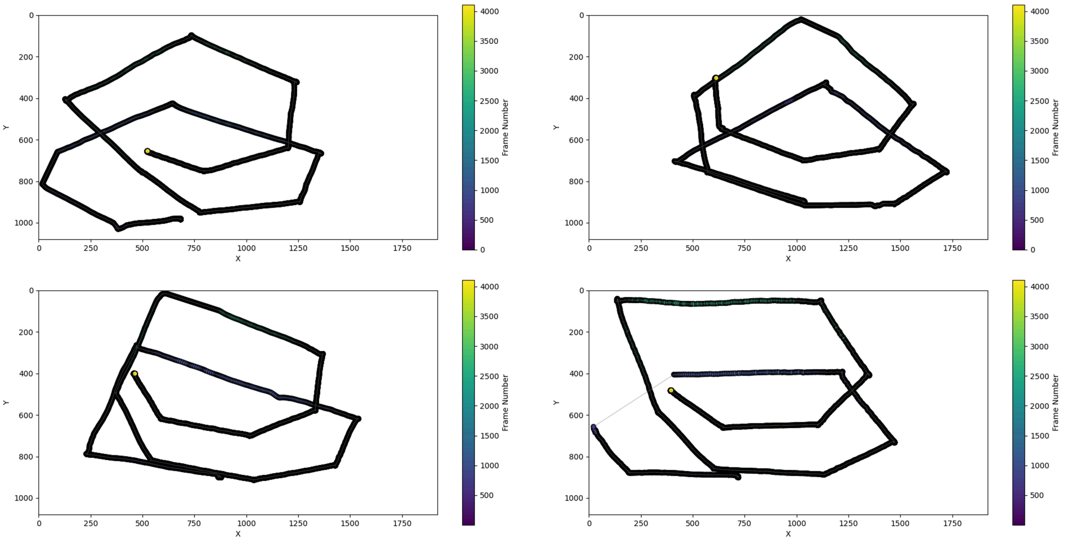

2. Run `e_matrix.py` to estimate camera poses and reconstruct the 3D trajectory.

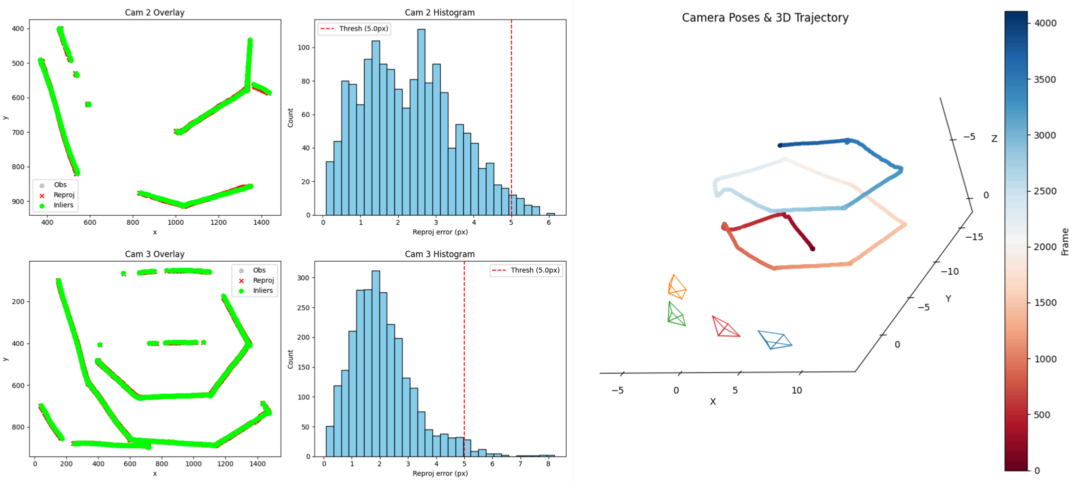

3. Run `align_gt.py` to align the reconstructed 3D trajectory to the GPS trajectory.

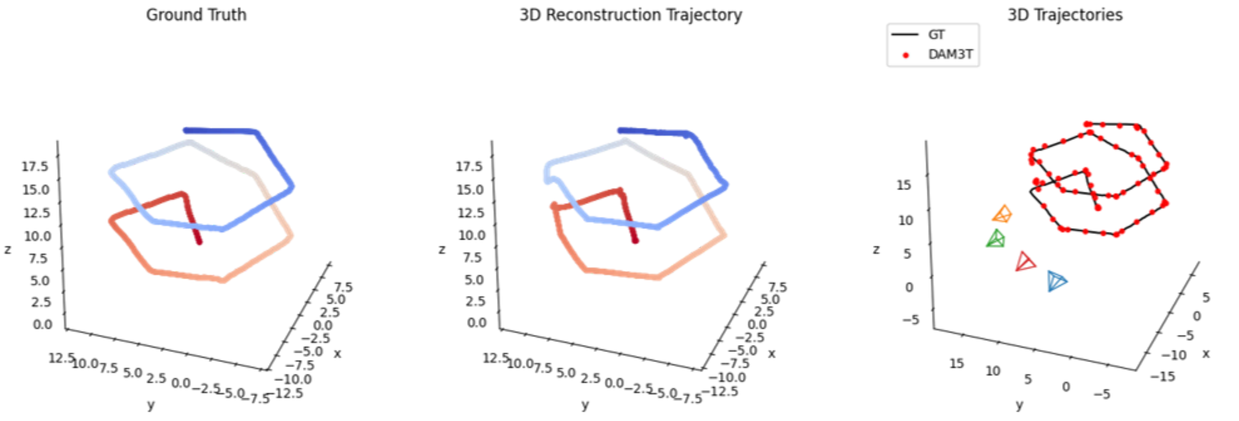

Multi-UAV Tracking

1. Use camera extrinsics obtained through the DAM3T workflow.
2. Prepare processed per-camera TXT detections for each target.
3. Run `ptv.py` to triangulate multi-target 3D trajectories. Optionally visualize the result and export KML.

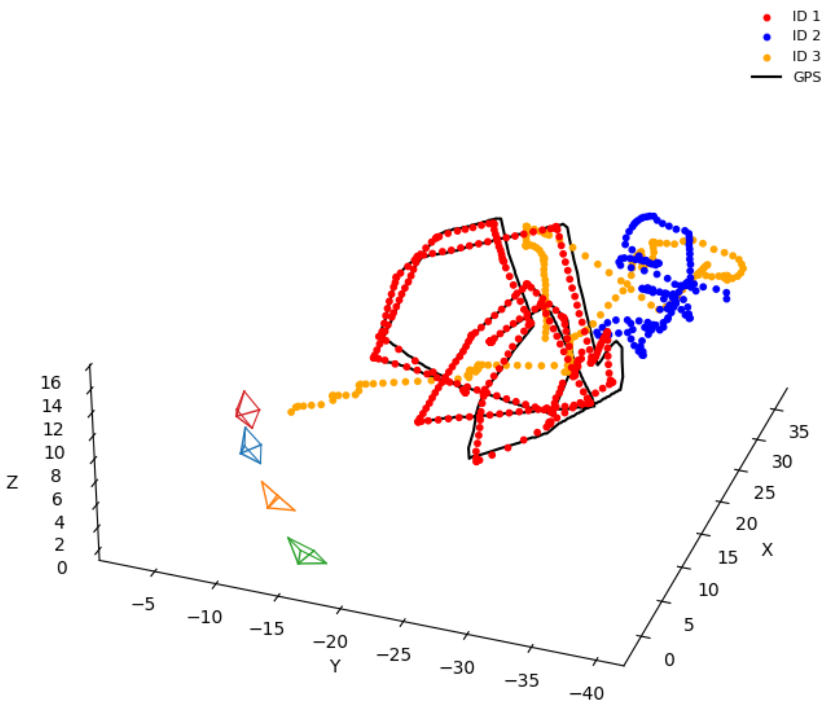

Folder Structure

```
.
├─ code/
│  ├─ 2d_tracking/
│  ├─ 3d_recon/
│  └─ visualization/
├─ gopro12/
│  ├─ calibration_images_fhd/
│  ├─ calibration_images_uhd/
│  └─ calibration_images_uhd_fhd/
├─ images/
├─ multi_uav_tracking/
├─ outdoor_validation/
│  ├─ cal_8/
│  ├─ cal_sp/
│     ├─ mot/
│     ├─ sot/
│     └─ vid/
│  └─ flight log/
├─ yolo/
└─ README.md
```

File Reference

`sot.py`

Single-object tracking with ROI initialization using OpenCV CSRT.

Input Variables

Variable	Type / Options	Description
`video_in`	`str`	Input video path
`txt_out`	`str`	Output TXT path for tracked center points
`video_out`	`str`	Output video path
`save_video`	`bool`	Save tracking result video
`max_display_width`	`int`	Resize width for interactive display

Output

Output	Description
`camX.txt`	Per-frame 2D trajectory in `frame x y` format
tracking video	Optional video with bounding box overlay

`mot.py`

Multi-object tracking using YOLO, ByteTrack, geometric filtering, and optional optical-flow-assisted box stabilization.

Using drone-specific YOLO weights from [Doguil Mak's repository].

Input Variables

Variable	Type / Options	Description
`video_in`	`str`	Input video path
`weights`	`str`	YOLO model weights
`video_out`	`str`	Output video path
`pkl_out`	`str`	Output PKL path
`save_video`	`bool`	Save tracking result video
`conf_th`	`float`	Detector confidence threshold
`imgsz`	`int`	Inference image size
`iou_th`	`float`	Detector IoU threshold
`max_det`	`int`	Maximum detections per frame
`class_filter`	`list[int]`	Allowed class IDs
`use_geom_filter`	`bool`	Enable bbox geometry filtering
`max_area_frac`	`float`	Maximum bbox area ratio to frame area
`min_area_frac`	`float`	Minimum bbox area ratio to frame area

Variable	Type / Options	Description
`min_ar`	`float`	Minimum bbox aspect ratio
`max_ar`	`float`	Maximum bbox aspect ratio
`show_window`	`bool`	Show debug window
`window_name`	`str`	Debug window title
`max_display_width`	`int`	Max display width
`use_optical_flow_assist`	`bool`	Fuse tracking with optical flow
`fuse_alpha`	`float`	Detection / flow fusion weight
`bbox_ema_beta`	`float`	EMA update factor for bbox smoothing
`gftt_max_corners`	`int`	Maximum corners for GFTT
`gftt_quality`	`float`	GFTT quality level
`gftt_min_dist`	`float`	Minimum corner spacing
`lk_win_size`	`tuple[int, int]`	Lucas-Kanade window size
`lk_max_level`	`int`	Lucas-Kanade pyramid levels
`lk_criteria`	`tuple`	Lucas-Kanade termination criteria
`min_good_pts`	`int`	Minimum good points for flow update
`max_median_speed_px`	`float`	Max median flow speed in pixels
`max_fb_err`	`float`	Max forward-backward optical flow error
`track_thresh`	`float`	ByteTrack score threshold
`track_buffer`	`int`	ByteTrack track buffer
`match_thresh`	`float`	ByteTrack matching threshold

Output

Output	Description
tracking PKL	Detection / tracking table saved as PKL
tracking video	Optional video with tracking overlays

`mot_post.py`

Post-processes the raw MOT PKL to extract a cleaned single-target 2D trajectory.

Input Variables

Variable	Type / Options	Description
`pkl_in`	`str`	Input MOT PKL from `mot.py`
`txt_out`	`str`	Output cleaned TXT trajectory
`show_vis`	`bool`	Show trajectory plot
`quality`	`str` : `fhd` , `uhd`	Resolution preset used for plotting and axis limits. `fhd = (1920, 1080)` , `uhd = (3840, 2160)`
`min_conf`	`float`	Minimum detection confidence
`max_bbox_area`	`float` or `None`	Maximum allowed bbox area
`static_grid`	`float`	Grid size for static background filtering
`static_min_hits`	`int`	Minimum hits to mark a static cell
`static_max_std`	`float`	Maximum std for static cell filtering
`static_exempt_track_len`	`int`	Exempt long positive tracks from static removal
`band_remove_enable`	`bool`	Enable band-background removal
`band_axis`	`str` : `x` , `y`	Axis used for band filtering

Variable	Type / Options	Description
`band_bin`	`float`	Band bin width
`band_min_hits`	`int`	Minimum hits to mark a bad band
`band_only_unassigned`	`bool`	Restrict band filtering to unassigned tracks
`min_anchor_len`	`int`	Minimum anchor length
`min_anchor_conf`	`float`	Minimum anchor confidence
`anchor_min_total_disp`	`float`	Minimum anchor displacement
`bg_track_aspect_th`	`float`	Background-track aspect ratio threshold
`bg_track_span_th`	`float`	Background-track span threshold
`anchor_connect_frame_gap`	`int`	Maximum frame gap between anchors
`anchor_connect_dist`	`float`	Maximum distance between anchors
`seed_search_radius`	`float`	Initial search radius for continuation
`step_gate`	`float`	Standard continuation gate
`step_gate_fast`	`float`	Fast-motion continuation gate
`lookback`	`int`	Lookback frames for velocity estimate
`bridge_max_gap`	`int`	Maximum interpolation / bridge gap
`continue_patience`	`int`	Allowed consecutive misses
`reacquire_max_gap`	`int`	Maximum gap for reacquisition
`edge_margin_ratio`	`float`	Edge margin ratio for tail trimming
`tail_low_conf_th`	`float`	Low-confidence threshold for tail cut
`tail_window`	`int`	Tail analysis window size
`tail_min_low_conf_ratio`	`float`	Minimum low-confidence ratio to trim tail
`smooth_window`	`int`	Smoothing window size

Output

Output	Description
cleaned TXT	Single-target trajectory in `frame x y` format
2D plot	Optional trajectory visualization

`mot_post2.py`

Alternative post-processing for fragmented MOT results. Builds a final trajectory by linking anchor tracklets and micro-tracklets.

Input Variables

Variable	Type / Options	Description
`pkl_in`	`str`	Input MOT PKL
`txt_out`	`str`	Output cleaned TXT
`show_vis`	`bool`	Show trajectory plot
`quality`	`str` : `fhd` , `uhd`	Resolution preset used for plotting and axis limits. `fhd = (1920, 1080)` , `uhd = (3840, 2160)`
`min_anchor_len`	`int`	Minimum anchor track length
`pos_max_frame_gap`	`int`	Maximum frame gap inside positive track segments
`pos_max_step`	`float`	Maximum spatial step inside positive tracks
`use_neg_only_for_micro`	`bool`	Use negative track IDs only for micro-tracklets
`micro_max_assign_dist`	`float`	Max assignment distance for micro-tracklets
`micro_dist_grow_per_skip`	`float`	Assignment growth per skipped frame

Variable	Type / Options	Description
`micro_max_skip`	`int`	Maximum skipped frames for micro-tracklets
`micro_min_len`	`int`	Minimum micro-tracklet length
`micro_min_motion`	`float`	Minimum micro-tracklet motion
`micro_min_span`	`int`	Minimum micro-tracklet span
`max_anchor_bridge_gap`	`int`	Maximum gap for anchor bridging
`max_transition_dist`	`float`	Maximum transition distance
`gap_penalty`	`float`	Gap penalty for DP linking
`dist_penalty`	`float`	Distance penalty for DP linking
`vel_penalty`	`float`	Velocity mismatch penalty
`min_bridge_gain`	`float`	Minimum gain for bridge acceptance
`max_side_gap`	`int`	Maximum side extension gap
`min_side_gain`	`float`	Minimum gain for side extension
`small_hole_interp`	`int`	Interpolate holes up to this size
`spike_gate`	`float`	Spike rejection threshold
`segment_min_keep_len`	`int`	Minimum valid segment length
`segment_island_max_len`	`int`	Maximum removable island length
`segment_bridge_max_gap`	`int`	Maximum gap for segment bridge evaluation
`segment_endpoint_dist_gate`	`float`	Endpoint consistency gate
`segment_line_deviation_gate`	`float`	Deviation-from-line threshold
`segment_jitter_ratio_gate`	`float`	Jitter ratio threshold
`segment_min_net_motion`	`float`	Minimum net motion for a valid segment

Output

Output	Description
cleaned TXT	Single-target 2D trajectory in `frame x y` format
2D plot	Optional trajectory visualization

`e_matrix.py`

Estimates camera poses and reconstructs a 3D trajectory from multiple cleaned 2D detection files.

Input Variables

Variable	Type / Options	Description
`json_in`	`str`	Camera intrinsic calibration JSON
`txt_in_folder`	`str`	Folder containing per-camera TXT tracks
`emat_out`	`str`	Output reconstruction PKL
`reproj_thresh`	`float`	Reprojection threshold in pixels
`min_required_views`	`int`	Minimum number of views for triangulation
`frustum_scale`	`float`	Camera frustum scale factor
`debug_plot`	`bool`	Show debug plots

Output

Output	Description
reconstruction PKL	Saved dictionary containing `cam_pose`, `cam_rotation`, `P_mats_cam0`, `3d_points`, `used_cams_per_frame`, `reprojection_summary`, `cam_file_map`, `post_alignment`, and `swap_keep_right_handed`
debug plots	Optional reprojection and 3D visualization plots

`align_gt.py`

Aligns the reconstructed 3D trajectory to the GPS trajectory using similarity transform and ICP-style refinement.

Input Variables

Variable	Type / Options	Description
`resample_num`	`int`	Number of resampled points used for alignment
`view_elev`	`float`	3D plot elevation
`view_azim`	`float`	3D plot azimuth
`frame_interval`	`int`	Visualization subsampling interval
`gps_in`	`str`	Input GPS PKL from `csv_to_pkl.py`
`e_mat_in`	`str`	Input reconstruction PKL from `e_matrix.py`
`fin_out`	`str`	Output aligned PKL

Output

Output	Description
aligned PKL	Reconstruction aligned to the GPS frame
transformed camera poses	Camera poses after similarity transform
metrics	RMSE, normalized RMSE, Chamfer-derived metrics, scale
comparison plots	Optional 3D comparison figures

`ptv.py`

Triangulates multiple target trajectories using known camera extrinsics.

Input Variables

Variable	Type / Options	Description
`json_in`	`str`	Intrinsic calibration JSON
`fin_in`	`str`	Input reconstruction / extrinsic PKL
`txt_in_folder`	`str`	Folder containing processed TXT detections
`gps_in`	`str`	Optional GPS PKL
`use_gps`	`bool`	Compare against GPS when available
`min_required_views`	`int`	Minimum number of views for triangulation
`vis_3d`	`bool`	Show 3D visualization
`vis_point_step`	`int`	Point subsampling step for visualization
`frustum_scale`	`float`	Camera frustum scale
`plot_frustum`	`bool`	Draw camera frustums
`kml_out_folder`	`str`	Output folder for KML export

Output

Output	Description
`trajs_by_pid`	Multi-target 3D trajectories keyed by target ID
3D plot	Optional visualization of trajectories, cameras, and GPS
KML file	Exported trajectories and optional GPS path in WGS84

`csv_to_pkl.py`

Converts DJI flight-log CSV data into a PKL trajectory.

Input Variables

Variable	Type / Options	Description
`csv_in`	`str`	Input flight-log CSV path
`out_pkl`	`str`	Output PKL path
`source_tz`	`str`	Source timezone of CSV timestamps
`target_tz`	`str`	Target timezone
`ref_mode`	`str` : `first` , `mean`	Origin selection mode. `first` uses the first valid GPS sample as origin; `mean` uses the mean GPS position
`frame_start`	`int`	Starting frame index
`frame_step`	`int`	Frame increment between consecutive GPS samples
`plot_3d`	`bool`	Show 3D visualization
`downsample_vis_n`	`int`	Number of points for visualization downsampling
`print_debug`	`bool`	Print debug messages

Output

Output	Description
GPS PKL	DataFrame containing `frame` , `x` , `y` , `z` , and optional `time_iso` , `local_time` , `time_s`
PKL metadata	Includes `origin_wgs84` , `ref_mode` , `crs` , and optional time-origin metadata

---

`vis_2d.py`

Plots a TXT trajectory in image coordinates.

Input Variables

Variable	Type / Options	Description
`txt_in`	`str`	Input TXT trajectory
`quality`	`str` : `fhd` , `uhd`	Resolution preset used for axis limits. `fhd = (1920, 1080)` , `uhd = (3840, 2160)`

Output

Output	Description
2D plot	Trajectory visualization

`cut_frame.py`

Utility script that replaces a specified frame range in a TXT trajectory with zero coordinates.

Input Variables

Variable	Type / Options	Description
`txt_out`	`str`	TXT file to edit
`frame_range`	`tuple[int, int]`	Inclusive `(start_frame, end_frame)` to overwrite

Output

Output	Description
modified TXT	Selected frames replaced with `0.00 0.00`
